# CRBN modulates synuclein fibrillation *via* degradation of DNAJB1 in mouse model of Parkinson disease

**DOI:** 10.1038/s41531-024-00801-3

**Published:** 2024-10-23

**Authors:** Uroos Akber, Jun-Hyung Jung, Heewoong Yoon, Jiwon Seo, Chul-Seung Park

**Affiliations:** 1 Laboratory of Molecular Neurobiology, School of Life Sciences, Gwangju Institute of Science and Technology (GIST), Gwangju, Republic of Korea; 2 Integrated Institute of Biomedical Research, Gwangju Institute of Science and Technology (GIST), Gwangju, Republic of Korea; 3 Department of Chemistry, Peptide Drug Discovery Laboratory, Gwangju Institute of Science and Technology (GIST), Gwangju, Republic of Korea

**Keywords:** Diseases of the nervous system, Cell biology, Chaperones

## Abstract

Cereblon (CRBN) is a substrate recruiter for CRL4^CRBN^ E3 ubiquitin ligase system playing a plethora of pivotal roles for biological systems. Here, we identified DNAJB1 (DJ1) as endogenous substrate of CRBN and report how CRBN influences the aggregation and toxicity of alpha-synuclein (α-SYN) *via* modulation of DJ1. CRBN interferes with molecular activities of DJ1 in vitro, in cells, and in vivo resulting in a reduced disaggregation of α-SYN fibrils, increased formation of preformed fibrils (PFFs) of α-SYN, and high susceptibility of mice to MPTP and PFF-induced neurotoxicity. Depletion of *Crbn* improves the behavioral and biochemical responses of mice towards neurotoxic insult. Finally, we designed a peptide inhibitor to inhibit the recruitment of DJ1 to CRBN for ubiquitination, resulting in an enhanced supply of DJ1 to counteract the toxicity of aggregated α-SYN. Our data has important implications for development of CRBN-targeting therapies that could prevent or delay progression of neurodegenerative synucleinopathy.

## Introduction

The CRL4^CRBN^ E3 ubiquitin ligase system is a macromolecular complex composed of Cullin-4 as the ligase scaffold, RING-finger protein RING-box1 as an adaptor for the E2 ubiquitin-conjugating enzyme, and Damage-specific DNA binding protein 1 as an adapter for substrate receptors such as Cereblon (CRBN)^1^. Because of its ability to recruit not only endogenous substrates naturally but also neosubstrates in the presence of immunomodulatory drugs (IMiDs), CRBN plays a plethora of roles requisite for biological systems. Examples of the endogenous targets of CRBN and their effects on cellular function include the actions of AMPK and glutamine synthetase on cellular metabolism^2,3^, Casein kinase 1α on cellular Wnt signaling^4^, redox proteins on cellular stress responses^5^, and ion channels on cellular excitability^6,7^. We recently reported the potential effects of CRBN on tauopathies via regulation of recruitment and degradation of the 70-kDa heat shock protein (HSP70) and DNAJA1 (DJ2)^8^. Neurodegenerative diseases such as Alzheimer’s disease (AD), Parkinson’s disease (PD), Huntington’s disease (HD), and amyotrophic lateral sclerosis (ALS) are multifactorial debilitating diseases of the nervous system involving misfolding and accumulation of aggregated proteins, mainly including tau and α-SYN. Despite extensive efforts to define the molecular mechanisms underlying neurodegeneration, several aspects of these disorders remain unknown. These elusive mechanisms represent a challenge to the characterization of viable drug targets and biomarkers for neurological disorders. Various cellular mechanisms linked to PD such as protein misfolding and aggregation, lysosome-autophagy impairment, proteasomal impairment, oxidative stress, reactive oxygen species imbalance, axonal mitochondrial dysfunction, calcium homeostasis, and neuroinflammation have been extensively studied^9^. The major cytopathological marker of PD is the formation of large cytoplasmic proteinaceous inclusions known as Lewy bodies that are predominantly composed of misfolded α-SYN^9^. α-SYN is plentifully expressed in neurons and encompasses 1% of the total cytosolic protein of brain cells^10^.

Proteins are fairly vulnerable in the physiological environment of cells. HSP70 plays a significant role in the maintenance of proteostasis in cells and is involved in a wide range of activities, such as protein folding, refolding of misfolded proteins, and targeting aggregated proteins for degradation^11^. HSP70 chaperones require the assistance of co-chaperones to perform their function. The DNAJ family of proteins is a major group of such co-chaperones, consisting of more than 40 different members^12^. Chaperones may also participate in the clearance of toxic aggregates in neurodegenerative diseases including PD. PD is highly correlated with large assemblies of α-SYN called amyloid fibrils (or preformed fibrils, PFFs), which comprise the main component of Lewy bodies. PFFs are challenging substrates for the cellular protein quality control system due to their highly ordered fibrillar and stable structure. Despite being extremely stable protein aggregates, amyloid fibers made of tau, huntingtin, or α-SYN can be broken down by a particular mix of human HSP70 system chaperones. HSC70, DNAJB1 (DJ1), and the nucleotide exchange factor APG2 are all necessary for disaggregation^13^. HSC70/DJ1/APG2 disaggregase generates sufficient force to break apart or depolymerize α-SYN amyloid fibers, leading to disaggregation^14^. Extensive research has been performed to examine therapeutic approaches to target chaperones involved in PD and restore the equilibrium of α-SYN. Recent advances in the study of AD, PD, HD, and ALS have suggested that numerous multifactorial degenerative processes contribute to neuronal death in neurodegenerative diseases, leading to functional impairments. Therefore, identification of novel potential biomarkers and new drug targets for neurodegeneration is necessary. In this study, we report CRBN as a measurable marker for the early diagnosis of PD due to its ability to fine-tune the in vitro and in vivo neuroprotective effects of DJ1 and DNAJB6 (DJ6) toward synucleinopathies induced by 1-methyl-4-phenyl-1,2,3,6-tetrahydropyridine (MPTP) and PFFs. Our results demonstrate that depletion or inhibition of CRBN can modulate α-SYN aggregation by sustaining the chaperone activities of DJ1 and DJ6 in brain cells.

## Results

### DJ1 and DJ6 are endogenous binding partners of CRBN

**Fig. 1:**
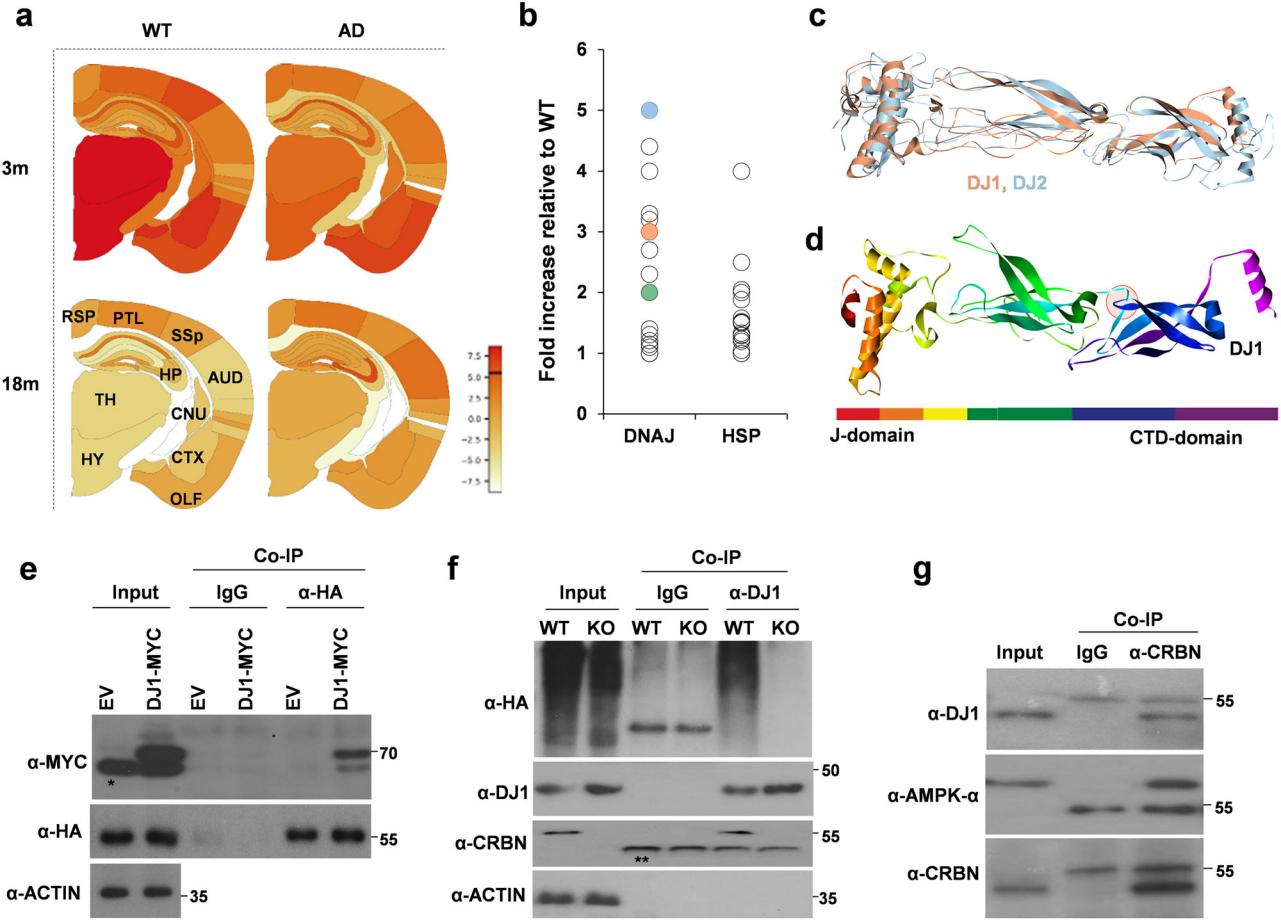
DJ1 is endogenous substrate of CRL4^CRBN^. **a** CRBN levels are high in the brains of AppNL-G-F mice, an AD mouse model as compared to wild type (WT) mice at the ages of 3 months (3 m) and 18 months (18 m). data was extracted from https://alzmap.org/ using “CRBN” as query. TH,thalamus; HYP,hippocampus; CNU,cerebralnucleus; CTXsp,corticalsubplate; OLF,olfactoryarea; ENTI,entorhinalarea; AUD,auditoryarea; SSp,primarysomatosensory; PLT,posteriorparietalassociationarea; RSP,retrosplenialarea. **b** Expression of certain chaperones (Heat shock proteins or HSP) and cochaperones (DNAJ proteins) is increased in the whole brain lysates of *Crbn*^-/-^ (KO) mice as compared to *Crbn*^+/+^ (WT) mice. DJ2, DJ1, and DJ6 are marked in blue, coral-red, and green, respectively. **c** DJ1 is structural homolog of DJ2. **d** β-turn- β loop of DJ2 involved in binding to CRBN is structurally conserved in DJ1. **e** CRBN binds to DJ1. SH-SY5Y cells were transiently co-transfected with HA-CRBN and Myc-DJ1* shows non-specific band, ** shows heavy chain band. Cells were lysed, immunoprecipitated with IgG control or α-HA antibody. *n* = 5 (**f**) DJ1 undergoes CRBN-mediated ubiquitination. SH-SY5Y cells were transiently transfected with HA-Ub and lysed in Ubn buffer, IP was performed with α-DJ1 antibody, *n* = 5. **g** CRBN binds to DJ1 in the brain lysates of mouse. Endogenous CRBN was pulled down with α-CRBN antibody and immunoblotting was performed with the antibodies indicated, AMPK-α was used as positive control.

Spatial transcriptomics analysis^15^ of AppNL-G-F mice, an AD mouse model, showed increased CRBN expression in most brain regions (Fig. 1a), which may interfere with the bioavailability and functioning of endogenous substrates of CRBN. We hypothesized that after knocking out CRBN, these endogenous substrates may exhibit increased abundance and thus improved bioactivity. In our previous report^8^, we showed that DJ2, a co-chaperone of HSP70, is an endogenous CRBN substrate, and its protein level is increased in the brain of *Crbn*^*-/-*^ (knockout, KO) mice. In addition to DJ2, co-chaperones of DNAJ proteins, including DJ1 and DJ6, were significantly increased in the brains of *Crbn*^*-/-*^ mice compared with those in *Crbn*^*+/+*^ (wild type, WT) mice (Fig. 1b). Both members of the DNAJB family exhibit structural homology with DJ2, and the β-turn-β loop involved in the binding to CRBN is also well conserved among these co-chaperones (Fig. 1c, d, Supplementary Fig. 1a–c). As expected, a coimmunoprecipitation (CoIP) assay showed robust binding of CRBN to DJ1 and DJ6 (Fig. 1e, Supplementary Fig. 2a), resulting in their ubiquitination (Fig. 1f, Supplementary Fig. 2b). These results strongly suggest that DJ1 and DJ6 are endogenous substrates of CRBN.

To determine if DJ1 and DJ6 bind to CRBN together as a complex or independently, we knocked down DJ6 in SHSY5Y cells and performed a CRBN pull-down assay. DJ1 was found to bind CRBN independently of D6 (Supplementary Fig. 2c). Based on observed higher expression levels of DJ1 compared to DJ6 (Fig. 1b), we opted to focus on DJ1 as the primary protein of interest for subsequent investigations. Next, to confirm the recruitment of DJ1 by CRBN in vivo, we repeated the Co-IP assay using whole-brain lysates from C57BL/6 mice and obtained the same result (Fig. 1g). It is worth noting that proteasomal ubiquitination of DJ1 was not affected by thalidomide, an IMiD that binds to CRBN and alters the associated ubiquitin ligase activity (Supplementary Fig. 2d). Using mouse embryonic fibroblast (MEF) cells, a delayed degradation of DJ1 was observed in the presence of cycloheximide (CHX) in *Crbn*^-/-^ MEF cells (Supplementary Fig. 2e).

### CRBN interferes with the folding activities of DJ1

**Fig. 2:**
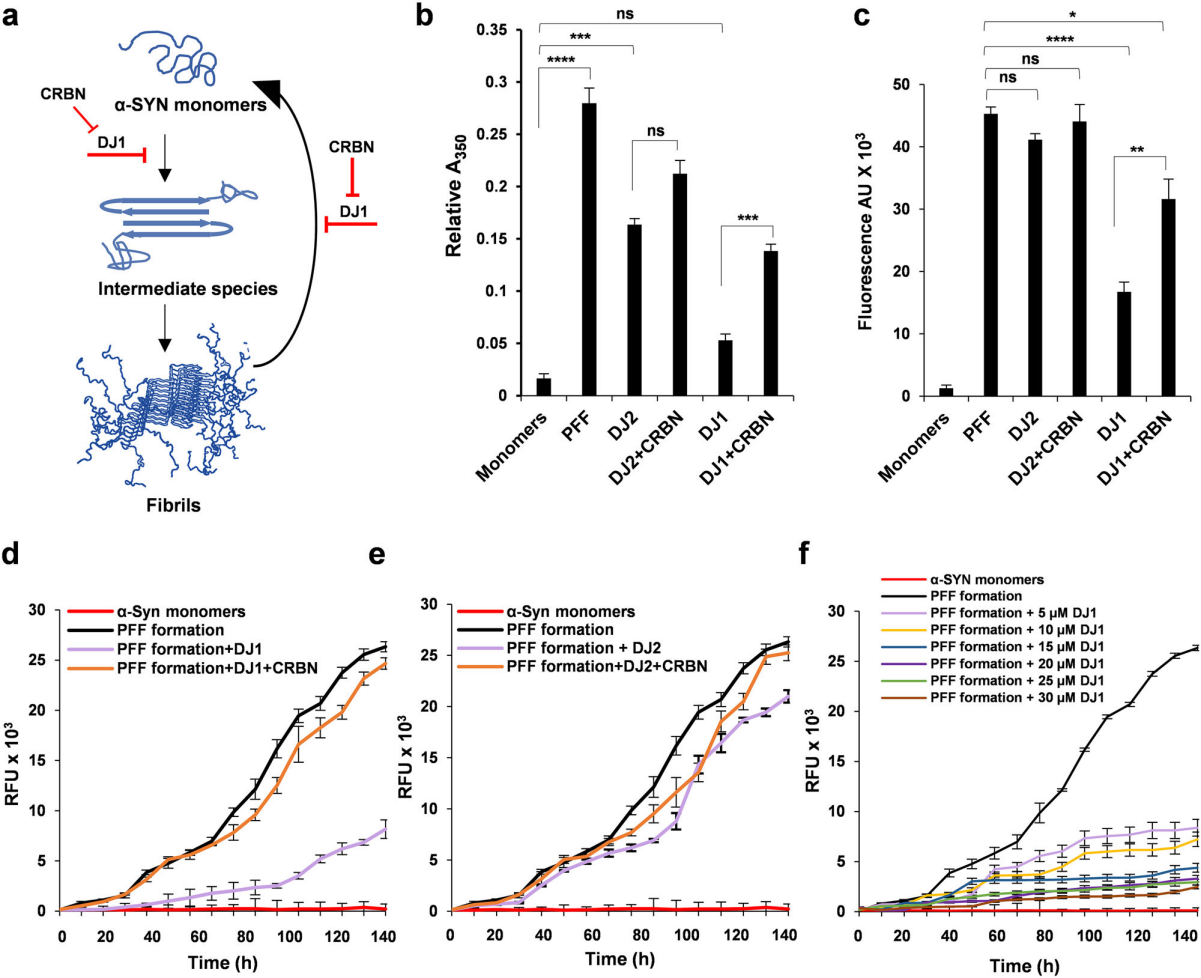
CRBN interferes with the chaperonic activities of DJ1. **a** Schematic representation of aggregation and disaggregation of α-SYN (adapted^32^). **b** Turbidimetry assay of α-SYN aggregates (prepared from 30 mg/mL purified recombinant α-SYN protein) incubated with or without 15 μM of DJ2, DJ1, and CRBN as mentioned (with 30 μM Hsp70, 15 μM HSPA4 or (APG2) and 2 mM ATP added to all the samples) and monitored at 500 nm. **c** Disaggregation assay of α-SYN aggregates. ThT fluorescence of α-SYN fibrils (2 μM) in the presence of chaperones (1 μM DJ1, DJ2, or CRBN as mentioned with 2 μM Hsp70, 1 μM APG2 and 2 mM ATP added to all the samples, incubated at 30 °C for 2 h. **d**, **e** Aggregation assays for α-SYN monomers incubated with mentioned combinations with 2 μM Hsp70, 1 μM APG2, and 2 mM ATP added to all the samples, incubated at 30 °C for 150 h with gentle rotatory shaking. **f** Aggregation assays for α-SYN monomers as mentioned in (**e**), performed in the presence of different concentrations of DJ1 indicated in the legend. Student’s *t* test and one-way ANOVA was used for the statistical analysis of data. Experiments were repeated 3 times with 3 samples (*n* = 9) for all data points. Significance levels (*p* values) are shown in Supplementary Table 1.

The functional significance of DJ1 in neurodegeneration has previously been reported^16,17^. We posited that CRBN can interfere with the chaperone activities of DJ1, resulting in rapid progression of α-SYN aggregation in vitro and in vivo. To validate this hypothesis, we measured the optical density of α-SYN aggregates synthesized with different combinations of chaperones. Fibrillogenesis of α-SYN changes its conformation from a predominantly unfolded form to a substantial β-structure^18^, referred to as a PFF (Fig. 2a). As shown in Fig. 2b, DJ1 but not DJ2, hampered the fibrillogenesis of α-SYN monomers. Addition of CRBN together with DJ1 significantly increased PFF formation presumably by sequestering the chaperone and thus affecting the chaperone-mediated reduction of aggregation and disassembly of α-SYN. Next, the fluorescent probe thioflavin T (ThT) was used to confirm the interference of CRBN in α-SYN disaggregation (Fig. 2c). PFFs were incubated with different combinations of chaperones (PFF, DJ2, and DJ2 + CRBN, DJ1 and DJ1 + CRBN) to determine the disaggregation capacity of DJ1 in the presence and absence of CRBN. As shown in Fig. 2c, CRBN significantly decreased the disaggregation of PFF. The same results were reproduced in the opposite assay through time-dependent ThT fluorescence, showing decreased aggregation of α-SYN monomers in the presence of DJ1but not DJ2 (Fig. 2d, e). However, a significant increase in α-SYN monomer aggregation was observed when CRBN was added with the chaperones to the aggregation mixture (Fig. 2d, e). To measure the chaperone capacity of DJ1, we repeated the aggregation assay using a concentration gradient of DJ1 and determined PFF formation. Here, a gradual increase in DJ1 concentration increased the chaperone activity of DJ1 and reduced the formation of PFF (Fig. 2f). Unfortunately, DJ1 concentrations above 30 µM resulted in reaction mixture aggregation. As such, we were not able to reach the saturation point needed for complete loss of PFF formation. To exclude the effects of CRBN and chaperones on ThT fluorescence, a control experiment was conducted to measure the fluorescence of individual proteins (Supplementary Fig. 3a, b). These results confirm the strong impact of CRBN on the anti-aggregation activities of DJ1.

### CRBN affects the toxicity of α-SYN fibrils

**Fig. 3:**
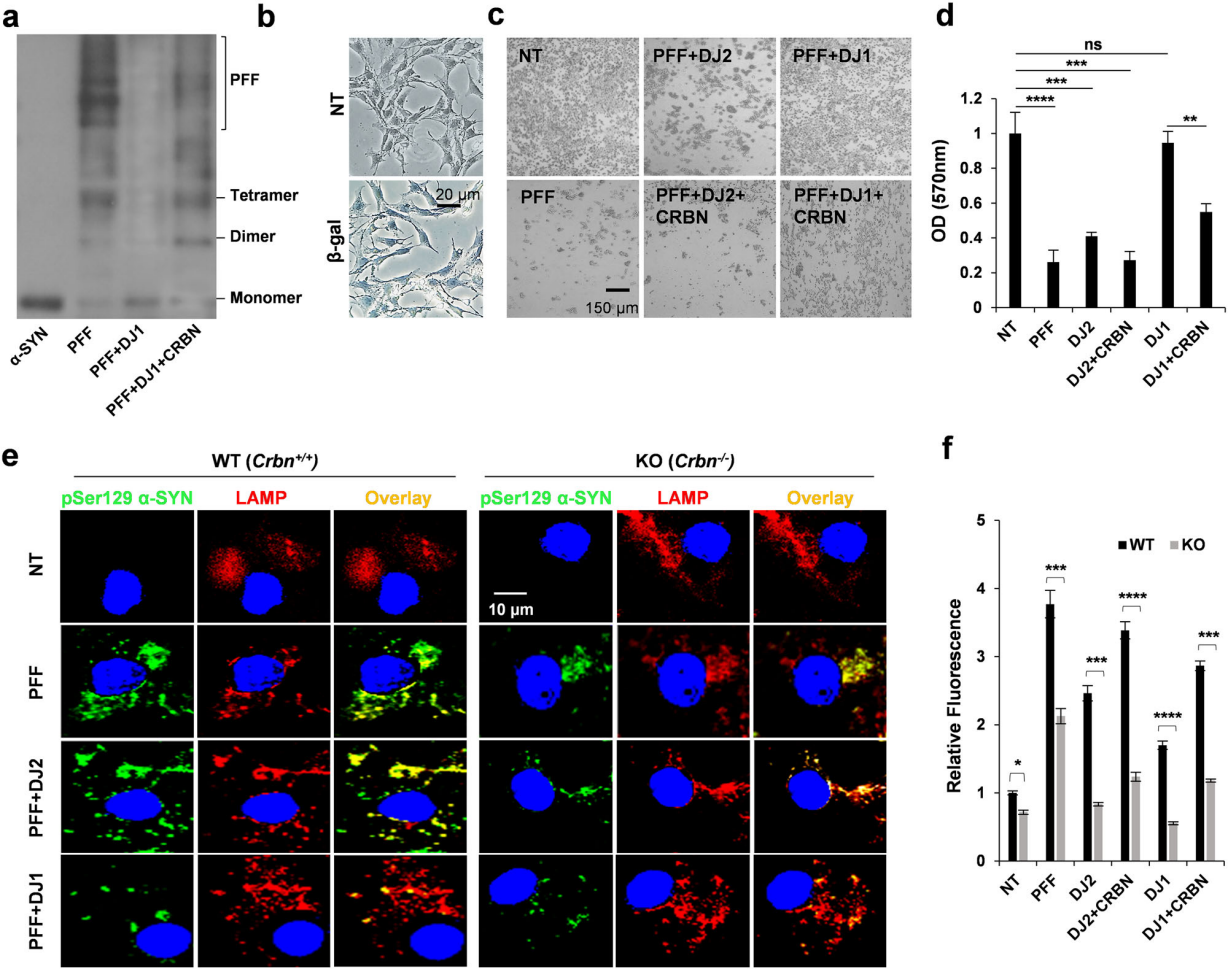
DJ1 decreases the toxicity of PFF in CRBN-dependent manner. **a** Western blot analysis of migration patterns of α-SYN monomers (1st lane) and PFFs generated without DJ1 (2nd lane), with DJ1 (3rd lane) and with DJ1 and CRBN (4th lane). **b** Differentiated SH-SY5Y cells were transfected with β-gal using Chariot^TM^ reagent for protein transfection as positive control to confirm the transfection efficiency. **c** Differentiated SH-SY5Y cells were treated with monomeric (named as non-treated NT) or PFFs prepared with or without DJ2, DJ1, and CRBN as mentioned in the legend of Fig. 2. **d** For the samples mentioned in (**c**), MTT assay was performed after 48 h to assess cell viability. **e** Differentiated SH-SY5Y cells were transfected with Myc Synuclein and re-transfected with PFFs prepared with or without DJ2, DJ1, and CRBN using Chariot^TM^. Cells were immunostained with pSer129 antibody (pSYN) and LAMP (lysosomal marker) and analyzed with confocal microscope after 48 h. Quantification is shown in the (**f**) panel. Error bars represent the SEM. Student’s *t* test was used for quantification of data with a 95% significance level in Excel. *n* = 5, *p < 0.05, **p < 0.01, and ***p < 0.001.

The size variation and morphology of fibrillar α-SYN have been closely associated with neuronal death in PD^19^. To measure the heterogeneity and corresponding toxicity of the PFF species used in this study, we first analyzed the migration patterns of the PFFs generated with different protein combinations. As expected, heavy molecular aggregates were nearly absent when α-SYN was incubated with DJ1 (Fig. 3a, 3rd lane). Next, we differentiated SH-SY5Y cells into dopaminergic-like neurons and transfected them with different PFF species using the protein transfection reagent Chariot^TM^ (Fig. 3b). Transfection of 30 μg/ml of PFFs into the differentiated dopaminergic-like neurons induced extensive cell death after 3 days (Fig. 3c, left column), while this effect was absent in α-SYN PFFs prepared with DJ1 (Fig. 3c, right upper panel). CRBN increased the toxicity of PFF species when added with DJ1 (Fig. 3c, right lower panels). Cell viability was also monitored for the same cells using an MTT assay, and identical results were obtained (Fig. 3d). Control samples were also analyzed to exclude interference of CRBN and chaperones while monitoring PFF toxicity (Supplementary Fig. 3c). These results further confirm that the purified CRBN protein can antagonize the anti-cytotoxic effects of DJ1 and strongly suggest that CRBN directly interacts with DJ1 and prevents its disaggregation activity.

### CRBN promotes the pathological phosphorylation and aggregation of α-SYN

We next sought to examine the effects of CRBN on α-SYN PFF toxicity in a cell-based system. WT and KO SH-SY5Y cells differentiated into dopaminergic-like neurons were transfected with Myc-synuclein. Both cell lines were treated with exogenous α-SYN monomers or the different PFF species mentioned above at a final concentration of 100 nM for 48 h. Only phosphate-buffered saline was added to non-treated (control) cells. Immunofluorescence examination of the cells revealed the formation of α-SYN inclusions in WT cells visualized by p-Ser129 α-SYN positivity (Fig. 3e, left panel), confirming seeding of endogenously expressed α-SYN by PFFs. Compared to WT cells, the formation of α-SYN inclusions was significantly reduced in CRBN KO cells (Fig. 3e, right panel). Intriguingly, PFFs generated in the presence of DJ1 induced markedly fewer α-SYN inclusions than did PFFs with α-SYN alone, even in WT cells (Fig. 3e, left-third panel). These effects were minimal in KO cells (Fig. 3e, right-third panel). The presence of CRBN in PFFs exaggerated the formation of α-SYN inclusions. These results show that CRBN affects the functionality of DJ1 not only in vitro but also in cells. Figure 3f shows the quantification of p-Ser129 α-SYN shown in Fig. 3e.

### *Crbn* KO causes cells to be resilient to α-SYN pathology

**Fig. 4:**
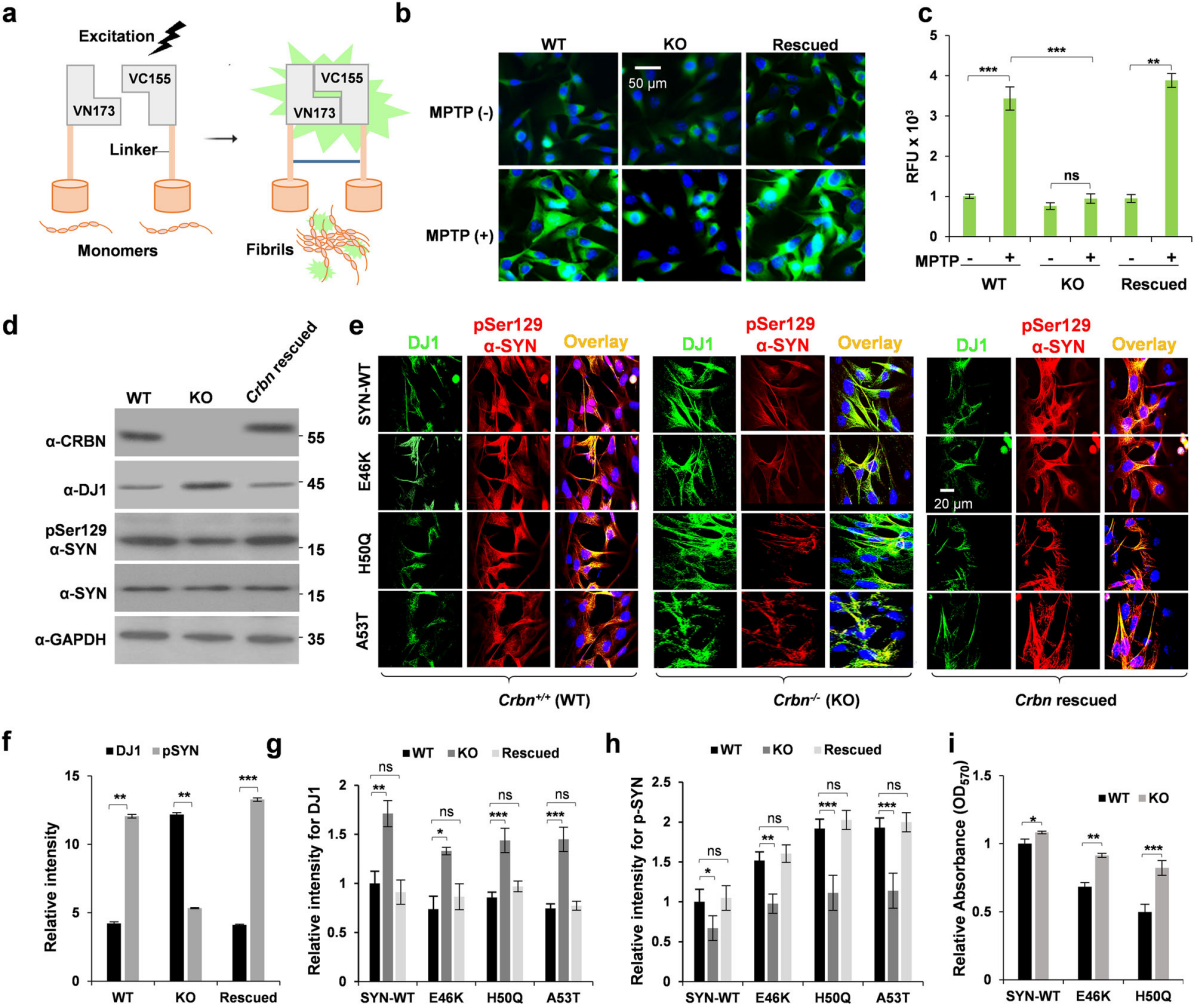
*CRBN*^*-/-*^ (KO) cells exhibit resistance toward the toxicity of α-SYN mutants. **a** Schematic representation of the Bimolecular Fluorescence Complementation (BiFC) assay. α-SYN is attached to non-fluorescent N- or C-terminal fragment of Venus fluorescence protein (VN173 or VC155). The Venus fluorescence turns on only when the phosphorylated tau assembles. **b**, **c** Effects of CRBN on phosphorylation-mediated α-SYN dimerization. Differentiated SH-SY5Y cells [*CRBN*^*+/+*^ (WT), *CRBN*^*-/-*^ (KO) and *CRBN*^*-/-*^ transfected with FLAG-CRBN (rescued)] were transfected with BiFC- Syn constructs and treated with 100 μM MPTP for 48 h. The cells were then imaged with an FV1000 confocal laser-scanning microscope. *n* = 5. **d** SHSY5Y cells mentioned in (**b**) were transfected with wild type (WT) or mutant forms of α-SYN as mentioned. DJ1 and p-Synuclein levels were measured by immunocytochemistry after 48 h. The cells were imaged with an FV1000 confocal laser-scanning microscope. *n* = 5. **f**–**h** Statistical analysis of (**d**, **e**) panels, pSer129 α-SYN of (**d**) is briefly written as pSYN in (**f**). **i** MTT assay was performed for the cell samples prepared in (**e**). Student’s *t* test was used to calculate *p* value for *n* = 6. Student’s *t* test and one-way ANOVA was used for the statistical analysis of data.

After examining the inhibitory effects of CRBN on DJ1 in vitro, we next further evaluated the consequent effects of CRBN in the cellular context. A bimolecular fluorescence complementation (BiFC) assay was used to monitor α-SYN oligomerization (Fig. 4a). SH-SY5Y (*Crbn* WT and KO) cell lines were differentiated into dopaminergic-like neurons and transfected with pBiFC-VN173-Syn and pBiFC-VC155-Syn vectors. The cells were treated with the neurotoxin MPTP to generate a cellular model of PD. BiFC signals induced by α-SYN oligomerization were significantly higher in WT cells than in KO dopaminergic-like neurons, and was significantly increased when *Crbn* was rescued back by overexpression of FLAG-CRBN in the KO dopaminergic-like neurons (Fig. 4b, c). Overexpression of CRBN and phosphorylation of α-SYN was also monitored by western blot analysis of these cells (Fig. 4d, f). These results were further confirmed by transfecting SH-SY5Y cells (*Crbn* WT and KO) with Myc-synuclein and treating them with MPTP. Pathological phosphorylation of Myc-synuclein was significantly reduced in KO cells with concomitant high expression of DJ1 (Supplementary Fig. 4a). We further investigated the effects of *Crbn* depletion on the enhanced pathogenicity of PD-linked mutations of α-SYN, E46K, H50Q and A53T. These mutants enhance α-SYN fibrillation^20^ and phosphorylation at Ser129^21^, which are pathological hallmarks of PD. *Crbn* WT and KO dopaminergic neurons were transfected with α-Syn WT, E46K, H50Q and A53T constructs, and phosphorylation of Ser129 was measured by confocal microscopy. As expected, KO neurons were resilient to the pathological impact of α-SYN E46K, H50Q and A53T mutants, as shown by reduced phosphorylation of Ser129 compared to that in WT neurons (Fig. 4e, g and h). The same samples were also observed by fluorescence microscopy to exclude any voltage-dependent effects of confocal microscopy (Supplementary Fig. 4b). An MTT assay was also performed for a sample identical to that shown in Fig. 4e. KO cells showed enhanced viability when transfected with pathological mutants of α-SYN (Fig. 4i). Increased DJ1 expression in the KO cell line may account for this resilience. Immunoblotting analysis demonstrated a high DJ1 level and low level of p-Ser129 α-SYN in the KO cell lines compared with those in the WT line (Supplementary Fig. 5a–c).

### *Crbn* KO has a positive impact on chaperones in vivo

**Fig. 5:**
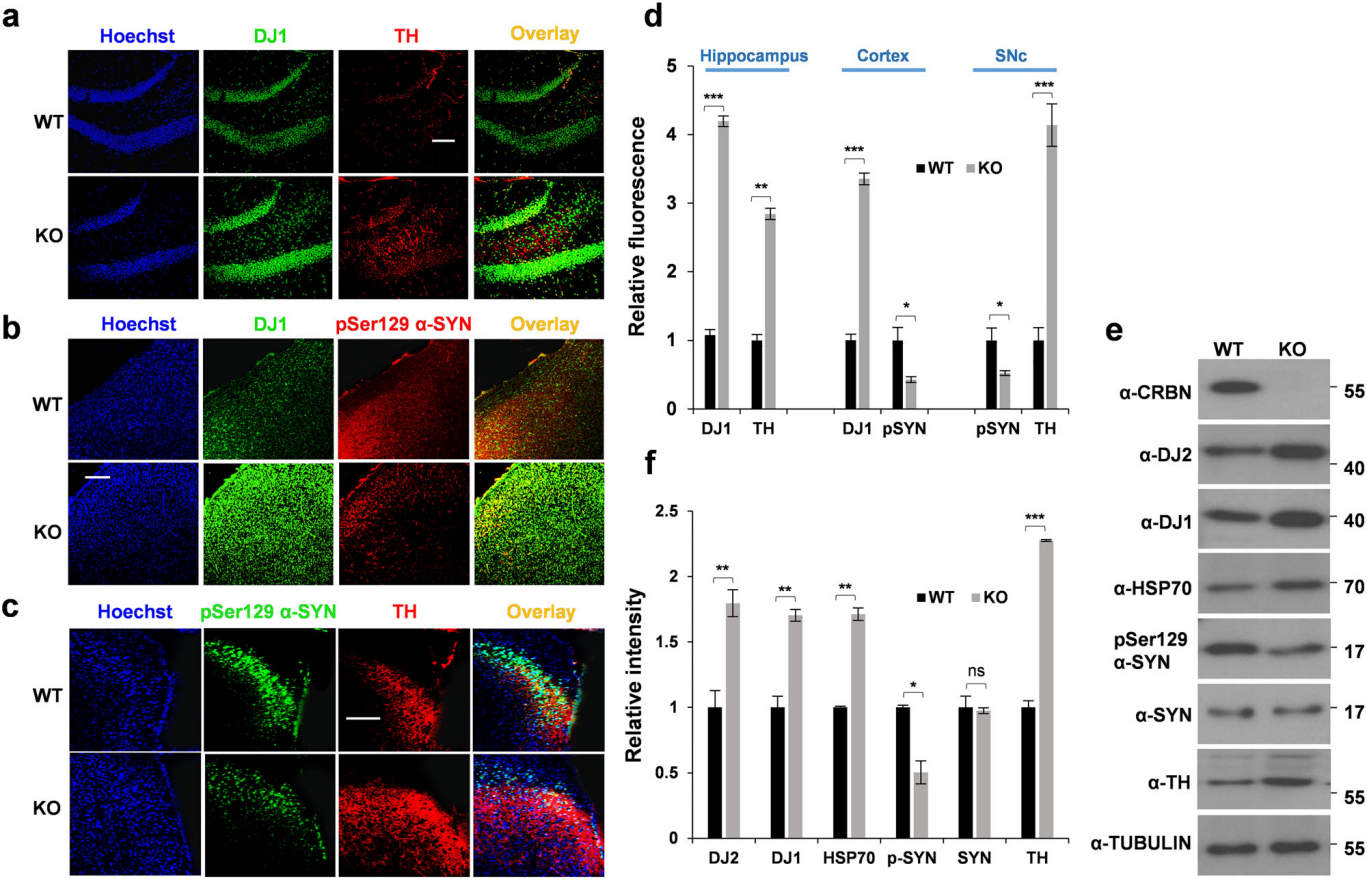
*Crbn*^*-/-*^ (KO) mice show abundance of DJ1 and TH. **a**–**c** Brains were removed from WT and KO mice, cryosectioned (25 µm thick) and immunostained with the antibodies mentioned for hippocampus (**a**), cortex (**b**) and Substantia Nigra pars compacta (SNc), scale bar = 100 µm. **d** Quantification of (**a**–**c**). **e**, **f** Brains were removed from WT and KO mice, lysates were prepared in RIPA buffer and immunoblotting was performed with the antibodies mentioned.

To validate our in vitro findings and assess their physiological relevance, we next performed in vivo experiments using *Crbn*^*+/+*^ (WT) and *Crbn*^*-/-*^ (KO) mice. Immunohistochemical staining of brain cryosections from 10-month-old WT and KO mice showed an increase in DJ1 chaperone expression with reduced phosphorylation of α-SYN at Ser129 (Fig. 5a–d). Because the loss of tyrosine hydroxylase (TH) is a co-phenomenon and driving factor for neurodegeneration in PD, we also performed TH immunochemistry. Interestingly, basal TH expression was increased in the hippocampi of *Crbn* KO mice (Fig. 5a, c). This result was also confirmed by immunoblotting (Fig. 5e, f), suggesting improved resistance to neurological insults in *Crbn* KO mice. Control samples without primary antibodies were prepared for several immunostaining samples throughout this study. Supplementary Fig. 6a shows control samples without primary antibodies in the granular region of the hippocampus. The hippocampus was immunostained with or without α-DJ1 antibody and counterstained with Hoechst; images were taken at 100x magnification to ensure that the overlapping confocal signals were not confounded by laser leakage. To amplify the signals of fluorescence for p-Ser129 α-SYN in Fig. 5b, c, laser intensity was slightly increased (Supplementary Fig. 6b). The magnified images were also taken at 100X (Supplementary Fig. 6c) to make sure that the immunostaining is consistent for WT and KO samples rather than being parenchymal or intracellular for different samples.

### *Crbn* modulates the neuroprotective capacity of mice

**Fig. 6:**
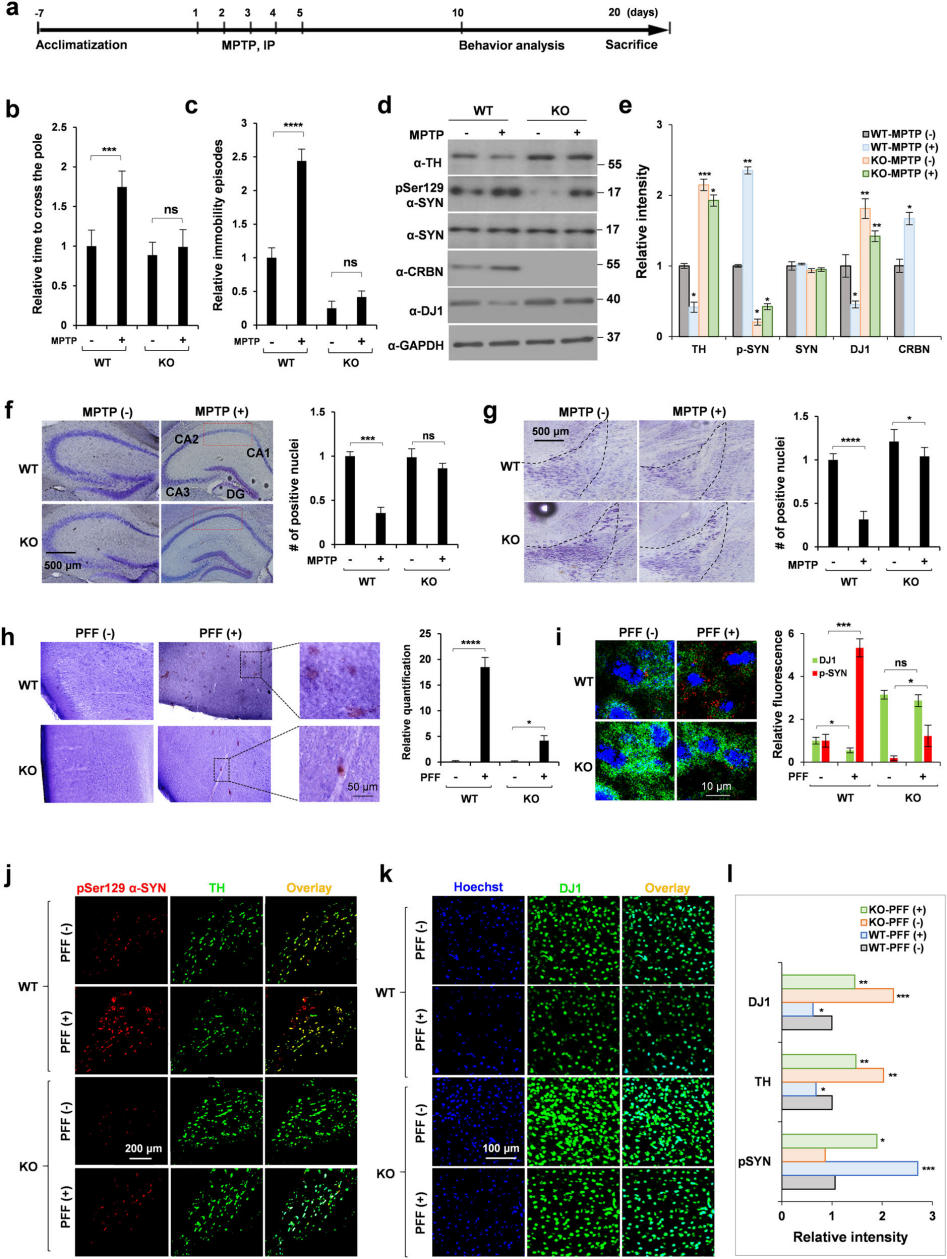
*Crbn*^*-/-*^ mice exhibit resistance towards MPTP- and PFF-induced parkinsonism. **a** Schematic representation of MPTP treatment paradigm. **b**, **c** Scoring of pole test and tail suspension test for WT and KO mice injected stereotaxically with human WT PFFs (5 μL of 5 μg/μL). **d**, **e** Brains were removed from WT and KO mice treated with MPTP, lysates were prepared in RIPA buffer and immunoblotting was performed with the antibodies mentioned. **f**, **g** Nissl staining of hippocampus and SNc regions of WT and KO mice injected intraperitoneally with MPTP. CA1, CA2, CA3: cornu ammonis 1, 2, 3 respectively; DG: dentate gyrus. **h**, **i** IHC staining of cortex of brains of WT and KO mice injected stereotaxically with human WT PFFs, using DAB substrate for pSer129α-SYN in (**h**) and alexa-conjugated antibodies in (**i**). Crystal violet was used for counter staining. IHC staining of SNc (**j**) and cortex (**k**) regions of brains of WT and KO mice injected stereotaxically with human WT PFFs with quantification shown in (**l**). Alexa flour secondary antibodies were used to monitor the levels of TH, DJ1 and p-synuclein. Sections were imaged with an FV1000 confocal laser-scanning microscope. ImajeJ was used for quantification of ROI. Student’s *t* test and one-way ANOVA was used for the statistical analysis of data.

The biochemical roles of DJ1 in the disaggregation of α-SYN PFFs have been well studied^13,22^, and α-SYN plays a central role in the pathology of PD. Hence, we extended our study to measure the modulatory effects of CRBN on MPTP-lesioned mice, which are in vivo models of PD. Intraperitoneal injections of MPTP produced drastic behavioral and biochemical changes that were significantly shifted in KO mice. The experimental paradigm is shown in Fig. 6a. The pole climbing test was used to assess motor dysfunction induced by MPTP. Among MPTP-treated animals, the time taken by KO mice to descend the pole was significantly less than that of WT mice (Fig. 6b). Similarly, immobility episodes in the tail suspension test were markedly decreased in KO mice compared with WT mice (Fig. 6c). Western blot analysis of WT whole-brain lysates from mice treated with MPTP showed an increase in CRBN and pathological phosphorylation of α-SYN with a concomitant decrease in DJ1 and TH (Fig. 6d, e). Conversely, *Crbn* KO mice showed resilience toward MPTP-induced pathology at both the behavioral and biochemical levels (Fig. 6b–e, Supplementary Fig. 6d).

Crystal violet staining of the granular region of the hippocampus and substantia nigra showed a noticeable decrease in the neuronal count in WT mice, whereas KO mice were resistant to MPTP-induced neuronal loss (Fig. 6f, g). Next, WT and KO animals were stereotaxically injected with PFF, and cryosections of WT and KO brains were stained for α-SYN aggregates using pS129 antibody. Increased phosphorylation of Synuclein was observed in the cortex (Fig. 6h) and substantia nigra of WT mice (Fig. 6i). Phosphorylation of S129 for α-SYN was much smaller in KO mice (Fig. 6i). Furthermore, cryosections analyzed by immunohistochemical staining revealed an elevated DJ1 signal in the brains of KO mice even after MPTP treatment (Fig. 6j, k). Statistical analysis of for α-SYN is shown in Fig. 6l. These results demonstrated that CRBN can modulate the neuroprotective capacity in mouse brains.

### C-terminal extension of DJ1 is required for interaction with CRBN

**Fig. 7:**
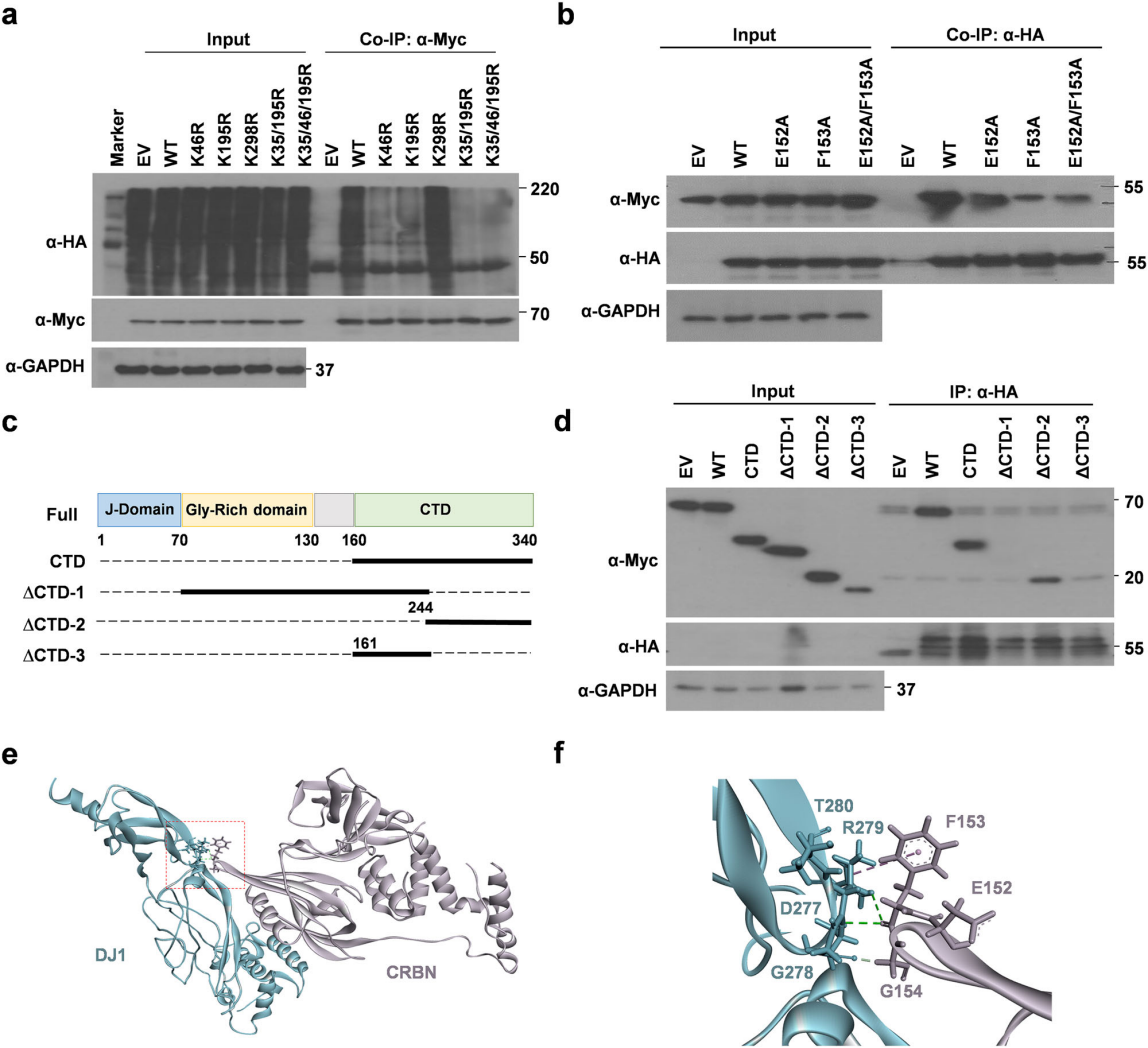
Localization of lysine residues responsible for the ubiquitination of DJ1 and residues responsible for binding to CRBN. **a** K35, K46, and K195 are the major ubiquitination sites in DJ1. SH-SY5Ycells were transfected with empty vector (EV), wild-type (WT) DJ1 or indicated lysine mutants. HA-Ub was co-transfected, cells were lysed in ubiquitination buffer, IP was performed with α-Myc antibody. *n* = 5. **b** E152 and F153 of CRBN are involved in the binding of CRBN to DJ2. SH-SY5Y cells were transiently co-transfected with Myc-DJ1 and the indicated constructs of HA-CRBN. After 24 h, cellular extracts were immunoprecipitated with α-Myc antibody. *n* = 5. **c** Schematic diagram of the deletion-mutant constructs of hDJ1 used in (**d**) with Myc-tag. **d** SH-SY5Y cells were transiently co-transfected with the genetic constructs indicated. After 24 h, cellular extracts were immunoprecipitated and blotted with the antibodies mentioned. *n* = 3. **e**, **f** Structural model of interaction of CRBN and DJ1 as predicted by ClusPro. PDB files of crystal structure of CRBN (4TZ4) and structural model of DJ1 generated by I-Tasser server were used. This binding mode was selected after combining the docking data with CoIP data.

Considering the pivotal role of CRBN in modulating α-SYN aggregation, we sought to identify the molecular determinants of DJ1 recruitment by CRBN. This information may be helpful in designing therapeutic methodologies to target CRBN-DJ1 interactions. To this end, we characterized the lysine residues of DJ1 targeted by CRL4^CRBN^ for ubiquitination. Lysine residues reported by PhosphoSitePlus^23^ were individually replaced with arginine, and a ubiquitination assay was conducted (Supplementary Fig. 7a). K35R, K46R, and K195R mutants exhibited compromised ubiquitination (Supplementary Fig. 7a). A triple mutant, K35R/K46R/K195R, showed almost complete loss of ubiquitination compared with that of WT DJ1 (Fig. 7a). Collectively, these findings suggest that these three lysine residues of DJ1 are the major sites targeted by CRL4^CRBN^ for ubiquitination and subsequent degradation.

Next, we identified the amino acid residues important for the interaction of DJ1 with CRBN. Because DJ1 is a structural homolog of DJ2, we were guided by our previous results regarding the interaction between CRBN and DJ2^8^. The CRBN deletion mutants described in our previous study were used to perform a CoIP assay with DJ1, which showed that the N-terminal domain of Lon-N (approximately 80 amino acids) in CRBN is critical for DJ1 binding (Supplementary Fig. 7b, c). Using a series of alanine mutants, we also identified that E152 and F153 are crucial, and replacing the two residues with alanine disrupted the interaction of CRBN with DJ1 (Fig. 7b). Similarly, we designed truncated DJ1 mutants (Fig. 7c) and performed pull-down assays. The segment consisting of amino acids 244 to 340 at the C-terminus was found to be crucial for interaction with CRBN (Fig. 7d). We then analyzed a structural model of DJ1 docked onto the crystal structure of CRBN using ClusPro^24^ (Fig. 7e). DJ1 binds to the same β-turn-β’ loop joining the β3 and β4 regions of CRBN via E152, F153, and G154. Intriguingly, critical interactions of DJ2 with CRBN were also conserved in DJ1 (Fig. 7f).

### The tetrapeptide DGRT masks CRBN to provide desired therapeutic effects

**Fig. 8:**
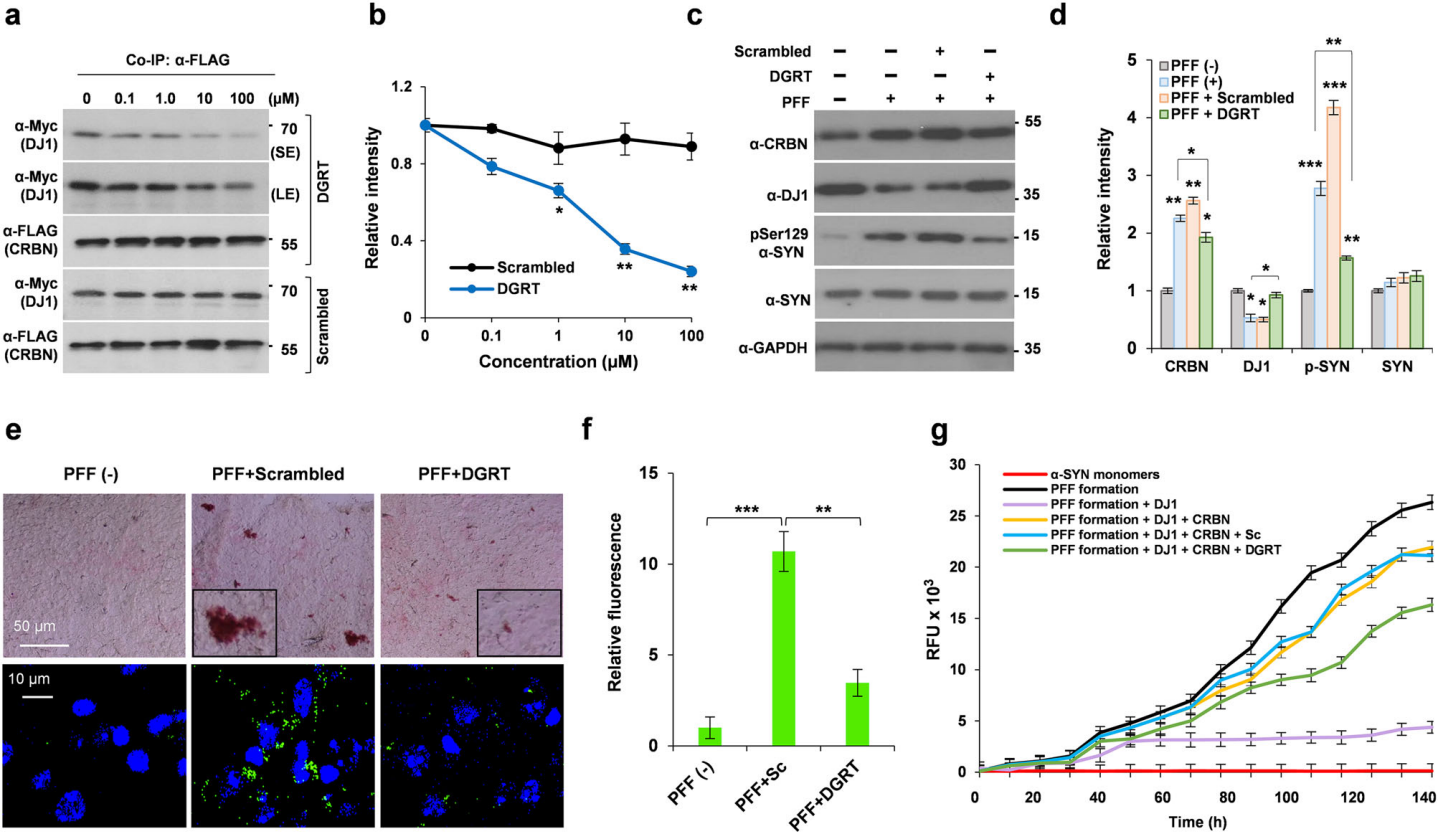
Bioactivity of tetrapeptide inhibitor to inhibit CRBN resulting in elevated DJ1 and reduced synuclein pathology. **a**, **b** SH-SY5Y cells were transiently co-transfected with Myc-DJ1 and FLAG-CRBN. Cells were lysed and incubated with scramble or DJ1-based tetrapeptide inhibitor DGRT with both ends blocked (Ac-DGRT-NH_2_). CoIP was performed with α-FLAG antibody. **c**, **d** WT mice were injected with PFF as described before alongwith the stereotaxic ICV injection of DGRT or scrambled peptide (10.3 μL of 10 mM stock to inject 50 μg). **e**, **f** IHC staining of substantia nigra of brain from WT mice using ImmPACT NovaRED^®^ substrate (upper panel) and alexa-conjugated antibody for pSer129 α-SYN staining. Data was analyzed by student’s *t* test and presented as mean ± SEM. Experiments were performed 6 times. *p < 0.05, **p < 0.01, and ***p < 0.001. **g** Aggregation assays for α-SYN monomers incubated with mentioned combinations with 2 μM Hsp70, 1 μM APG2 and 2 mM ATP added to all the samples, incubated at 30 °C for 150 h with gentle rotatory shaking. Ac-DGRT-NH_2_ inhibited the effects of CRBN as shown by green line.

Based on the hotspot region of DJ1 responsible for binding to CRBN, we synthesized a small tetrapeptide inhibitor composed of the following amino acid sequence of DJ1: D277, G278, R279, and T280. The N- and C-terminal regions were modified by acetylation and amidation, respectively. The peptide, Ac-DGRT-NH_2_, significantly inhibited the interaction between CRBN and DJ2 at a concentration of 10 µM, as shown by a CoIP assay (Fig. 8a, b). A scrambled peptide, Ac-RTDG-NH_2_, used as a positive control, did not significantly affect the binding of CRBN and D1. To test the neuroprotective effect of the tetrapeptide, we performed intracerebroventricular stereotaxic injections of PFFs (5 µg) into C57BL/6 WT mice along with the tetrapeptide or the scrambled peptide (50 µM). Animals were sacrificed after 30 days, and the brains were removed and processed for biochemical analyses. Western blot analysis of whole-brain lysate showed a significant elevation of CRBN and p-Ser 129 α-SYN, and a significant decrease in DJ1 after treatment with PFFs alone (Fig. 8c, d). However, all of these effects were reversed by co-injection with the Ac-DGRT-NH_2_ tetrapeptide (Fig. 8c, d). When the brain sections were immunostained with p-Ser 129 α-SYN. The anti-aggregation efficacy of the tetrapeptide was confirmed by a marked decrease of p-Ser 129 α-SYN staining in the samples treated with Ac-DGRT-NH_2_ peptide (Fig. 8e, f). Finally, we checked the inhibitory potency of the Ac-DGRT-NH_2_ peptide to minimize the effects of CRBN on α-SYN aggregation in vitro. Ac-DGRT-NH_2_ peptide inhibited CRBN not completely, but significantly in the in vitro aggregation assay (Fig. 8g).

A tetrapeptide based on the binding loop sequence of DJ6 (Ac-LLRH-NH_2_) was also synthesized, and its inhibitory activity on the recruitment of DJ6 to CRBN was monitored through CoIP and western blot analysis. Compared with the tetrapeptide based on the sequence of DJ1, a higher concentration of the DJ6-based tetrapeptide was required to interrupt the interaction between CRBN and DJ6 (Supplementary Fig. 8a). The scrambled peptide (Ac-RHLL-NH_2_) was used as a positive control. The synthetic scheme, high-performance liquid chromatography chromatogram, and electrospray ionization mass spectrometry spectra of the peptides used in this study are shown in Supplementary Figs. 9–10.

## Discussion

**Fig. 9:**
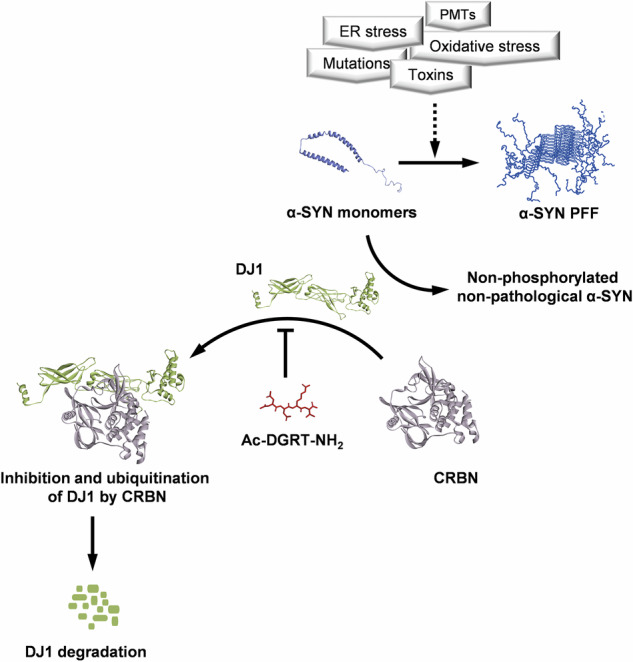
Schematic model of how CRBN modulates pathological forms of α-SYN. Several insults including oxidative stress can lead to an increase in the aggregation of α-SYN. DJ1 is a master regulator of the non-pathological states of α-SYN. Recruitment and degradation of DJ1 by CRBN decreases the availability of DJ1. CRBN-mediated modulation of chaperone activity of DJ1 disturbs the homeostatic balance, eventually triggering or promoting PD. Hence, inhibition of CRBN to recruit DJ1 may prevent the formation of oligomeric α-SYN by maintaining a non-phosphorylated and non-pathological pool of α-SYN. PDB ID for the 3D structure of α-SYN PFF is 2NOA, for α-SYN monomer is 1XQ8, for CRBN is 4CI3, and for DJ1 the 3D structure was modeled by I-TASSER server^33^. Structure of tetrapeptide inhibitor was drawn using ChemDraw software.

Accumulation of misfolded and aggregated proteins can be a source of neurodegeneration. α-SYN is predisposed to aggregate and form PFFs in the presence of high concentrations, oxidation, and inhibition of proteasomes^25,26^. DNAJ proteins are co-chaperones that facilitate the clearance of aggregated proteins and promote protein folding to counteract accumulation of aggregated proteins. In this study, we extended the therapeutic scope of CRBN-mediated activities of DJ1 by designing a peptide inhibitor that has potent activity against synuclein aggregation (Fig. 9). We initially identified and confirmed DJ1 as new endogenous substrate recruited by CRBN. Our mutational study showed that CRBN recruits DJ1 proteins through its N-terminal region, unlike other substrates described previously. Thus, binding and subsequent ubiquitination of DJ1 are not affected by IMiDs such as thalidomide. Recruitment and degradation of DJ1 facilitates the accumulation of misfolded and pathologically phosphorylated α-SYN; hence, synucleinopathies may undergo rapid progression. In contrast, masking the binding site for DJ1 on CRBN using a small peptide inhibitor decreases the recruitment and ubiquitination of DJ1, resulting in increased availability of cellular DJ1.

Several studies have previously reported the potential of DJ1 to prevent α-SYN misfolding and amyloid protein aggregation^17,27^. Specifically, DJ1 has been shown to suppress PFF aggregation when it is overexpressed in cells and to dissolve PFFs in vitro^17^. Here, we observed the chaperone activity of DJ1 in a CRBN-dependent manner not only through in vitro and cell-based assays, but also using in vivo models of PD. These findings add a new layer of complexity to the biology of amyloid aggregates. Thus, the parameters influencing α-SYN and DJ1 functionality in neurons should be taken into account when explaining the pathophysiology of PD, including coordination with other cellular components such as CRBN and other members of the chaperone network.

In the present study, we showed that CRBN regulates the abundance of DJ1 in cells. This co-chaperone is member of powerful disaggregase machinery. DJ1 can not only disassemble large α-SYN PFFs into less toxic fragments or even monomeric α-SYN species, but also contributes to delaying the onset of PD as shown by our in vivo study of PD models. Considering the high basal levels of DJ1, DJ2, DJ6, and TH in the cells and animals with a *Crbn* KO background (Fig. 5C), the underlying molecular mechanisms to protect against neurodegeneration and hallmarks of PD are comprehensible, further highlighting the beneficial potential of CRBN for management of PD symptoms. Certain α-SYN mutations (A30P, A53T, E46K, H50Q) are involved in familial PD and significantly accelerate α-SYN aggregation and amyloid formation^20^. Because oligomeric PFFs and fibril precursors of α-SYN are strong neurotoxins and potent pathogenic entities of PD, the ability of *Crbn* KO neurons to resist the pathogenicity of α-SYN mutations shows remarkable clinical potential. MPTP is widely used as a neurotoxin to generate PD models. MPTP treatment can markedly increase α-SYN mRNA and protein expression in the brains of MPTP-lesioned mice, and up-regulation of α-SYN contributes to the cascade of deleterious measures that ultimately cause neuron death in the substantia nigra^28^. We found that dopaminergic-like neurons and mice with a *Crbn* KO background were markedly resistant to MPTP-induced toxicity, as measured by MTT assays and pathological phosphorylation of α-SYN. Overexpression of certain chaperones and co-chaperones may collectively have a strong impact on the resistance of *Crbn* KO mice to synucleinopathies and tauopathies. For example, DJ6 decreases Huntingtin (polyQhtt) aggregation^16^, DJ2 counteracts tau aggregation in vitro and in vivo^8^, and DJ1 facilitates the disaggregation and unfolding of PFFs in vitro and in vivo. Hence, the resilience exhibited by *Crbn* KO mice and cell lines toward neurodegeneration and α-SYN pathology is remarkable, and a noticeable abundance of co-chaperones could be a primary driving force behind this effect. Hippocampal atrophy on a structural MRI is probably the best-documented specific pathological feature in neurodegeneration^29,30^. Therefore, we analyzed the expression of DJ1, TH, and pSer129 α-SYN in the hippocampal brain region. Interestingly, we found high expression of TH in the brains of *Crbn* KO mice. TH can either be an endogenous substrate of CRBN, hence is abundant in the brains of *Crbn*^*-/-*^ mice, or its level was found to be increased due to an increased number of neurons as shown by crystal violet staining. Our findings pave the way for future studies to investigate the molecular pathways contributing to the TH abundance.

Another important finding of this study is the effect of knocking out *Crbn* on mouse behavior. MPTP-treated WT mice showed severely impaired locomotion compared with that of *Crbn* KO mice. We recently reported the strong resilience of *Crbn* KO mice to chronic ultra-mild stresses^5^. Here, we report resistance of *Crbn* KO mice to MPTP-induced toxicity and thus PD pathology. Behavioral analyses showed that the absence of CRBN resulted in a significant neuroprotective effect against PD. The importance of chaperones and co-chaperones has been reported in mice experiencing increased proteotoxic stress^31^. To prevent misfolding and aggregation of proteins, the cellular demand for chaperones is increased in stressed/toxicant-treated cells that accumulate damaged proteins. The ability of CRBN to regulate molecular chaperones can be effective in alleviating the cellular burden and maintaining cellular proteostasis, suggesting its therapeutic potential in many neurodegenerative disorders. The outcomes of this study revealed that the ability of *Crbn* to target several endogenous substrates, such as DJ1, suggests a wide range of new therapeutic applications to alleviate neurodegeneration and PD. Sustained CRBN activity may impair the ability of cells to clear misfolded or aggregated proteins, leading to their accumulation and subsequent neurodegeneration. Understanding the precise mechanisms by which CRBN contributes to neurodegeneration is crucial for the development of targeted therapies to restore protein homeostasis and halt disease progression in affected individuals.

To sum up, our study has uncovered a novel molecular mechanism crucial for averting PD. Identification of pivotal roles of CRBN in PD makes a substantial contribution to the understanding of intricate pathways involved in the etiology of PD and provides a therapeutic approach for the development of targeted interventions. Based on this knowledge, we rationally designed a promising inhibitor that holds potential for the effective decline in the progression of PD. While future studies are needed to thoroughly assess the additional consequences of inhibition of CRBN, based on the current data, modulation of DNAJB1 by inhibiting CRBN represents a promising new therapeutic for PD.

## Materials and methods

### Experimental animals and cell lines

C57BL6 male mice (25–35 g, 3–3.5 months old, otherwise mentioned) were used in the present study. Mice were housed in groups of 5 animals per plastic cage, maintained on a standard chow diet and water *ad libitum* in pathogen-free conditions with a 12-h light-dark cycle. 5XFAD male mice expressing five mutations in human AβPP and PS1 (B6SJL-Tg [AβPP *K670N*M671L*I716V*V717I, PSEN1*M146*L286V] 6799Vas/J) were purchased from The Jackson Laboratory and kept under the same conditions as C57BL6 mice. Mice were randomly distributed into experimental groups. All experiments and paradigms were approved by the Gwangju Institute of Science and Technology Animal Care and Use Committee. Maximum efforts were made to minimize the number of animals used with reduced suffering. Human neuroblastoma cell line SH-SY5Y (Invitrogen) was maintained in Dulbecco’s Modified Eagle’s medium (DMEM; Invitrogen) supplemented with 10% fetal bovine serum (FBS; Invitrogen) and 100 U ml^−1^ antibiotic-antimycotic (Invitrogen) at 37 °C with 5% CO_2_. *CRBN* was knocked out from the cells using CRISPR/cas9 system.

### Reagents and antibodies

Reagents and antibodies used in this study are detailed in Tables 1 and 2, respectively.

**Table 1:** Reagents and peptides used in the study

REAGENT	SOURCE	CATALOG NUMBER
Thalidomide	Sigma	Cat# 50-35-1
IPTG	Sigma	Cat# 367-93-1
ThT	Sigma	Cat# 9041-08-1
Lipofectamine 2000	Invitrogen	Cat# 11668019
PMSF	Sigma	Cat# 10837091001
Protease inhibitor cocktail	Calbiochem	Cat# 535140
Normal goat serum	Abcam	Cat# Ab7481
Aquamount	Thermoscientific	Cat# TA-125-AM
Hoescht Dye	Abcam	Cat# 145597
FSC22 clear	Leica	Cat# 3801480
DAB substrate	Vector lab.	Cat# SK-4100
ImmPACT NovaRED®	Vector lab.	Cat# SK-4805
ProteinG Sepharose	GE Healthcare	Cat# 17-0618-01
Metal Affinity resin	Clontech	Cat# 635502
Flag M2 agarose beads	Sigma	Cat# A2220
MPTP	Sigma	Cat# 506382
Chariot^TM^	Activemotif	Cat# 30025
Recombinant protein α-SYN	This Study	NA
Recombinant protein DNAJB1	This Study	NA
Recombinant protein DNAJB6	This Study	NA
Recombinant protein DNAJA1	This Study	NA
Recombinant protein Hsp70	This Study	NA
Recombinant protein APG2	This Study	NA

**Table 2:** Antibodies used in the study

ANTIBODIES	SOURCE	CATALOG NUMBER
α-CRBN	Cell Signaling	Cat# 71810,
α-DJ1	Abcam	Cat # ab231577
α-DJ6	Abcam	Cat # ab198995
α-FLAG	CST	Cat # 2368S
α-FLAG	Sigma	Cat # F1804
α-HA	Santa Cruz	Cat # sc-7392
α-ACTIN	Sigma	Cat# A0483
α-GAPDH	AbFrontier	Cat # PA0018
α-Tubulin	Sigma	Cat # T6199
α-Myc	Millipore	Cat # 05-419
α–HSP70	Santa Cruz	Cat # sc-32239;
α-TH	Abcam	Cat # ab112
α-SYN	Abcam	Cat # ab1903
α-p-SYN	Abcam	Cat # ab184674
α-p-SYN	CST	Cat # 23706S
α-β-amyloid	Abcam	Cat # ab201062
Alexa-488 anti-mouse	Invitrogen	Cat # A11029
Alexa-488 anti-rabbit	Thermo Fisher	Cat # A11034
Alexa-594 anti-mouse	Life technologies	Cat # A11005
Texas Red anti-rabbit	Invitrogen	Cat # T6391

### Generation of PD mice models

*Crbn* WT and KO mice were intraperitoneally injected with MPTP (30 mg/kg) for 5 consecutive days, while the control group was injected with an equal volume of normal saline. Two days after the last injection, behavioral assessments were performed through the pole test and tail suspension test. On the 20th day, all animals were sacrificed for further study. For behavioral analysis, blind scoring was carried out by observers unfamiliar to the experiments.

### Immunoprecipitation (IP)

Cells were lysed in RIPA buffer (20 mM Hepes, 150 mM NaCl, 1 mM EDTA-EGTA, 1% Triton X-100, 1% NP40, 1% sodium deoxycholate, 2 mM Na_4_VO_3_, 100 mM NaF [pH 7.4]; supplemented with PMSF and protease inhibitor cocktail) or Tris buffer (20 mM Tris [pH 7.4], 0.32 M sucrose; supplemented with PMSF and protease inhibitor cocktail) followed by centrifugation at 12,500 RPM for 30 min. Proteins were immunoprecipitated with the specified antibodies for 16 h at 4 °C, followed by incubation with protein G Sepharose 4 Fast Flow for 2.5 h at 4 °C. Afterward, the beads were washed twice in RIPA or Tris buffer and twice in PBS. Immunoprecipitated proteins were resolved by SDS-PAGE, transferred to a PVDF membrane, and analyzed by immunoblotting with the specified antibodies.

### Transfection

1 × 10^5^ cells were seeded in 6-well plates and transfected with 500–1000 ng DNA the next day using Lipofectamine 2000 or Chariot^TM^ reagent for protein transfection. Negative control samples were transfected with Empty Vector (EV). Cells were harvested after 16 h for lysis or next step.

### Immunohistochemistry

The animals were euthanized, brain samples were fixed in 4% paraformaldehyde at 4 °C for 48 h, embedded in frozen section compound (OCT), and sectioned at 25 μm thickness. For antigen retrieval, the sections were placed in a citrate buffer (pH 6.0) and heated in an incubator at 90 °C for 45 min. Endogenous peroxidase activity was blocked by placing the sections in 0.3% H_2_O_2_ in methanol for 15 min at room temperature. Blocking was carried out for 1 h at 4 °C in blocking buffer (1% BSA, 10% goat serum in TBST, 0.3 M glycine). Sections were then incubated overnight at 4 °C with primary antibodies (1 : 1000), washed, and incubated with the corresponding (i) Alexa-conjugated secondary antibodies (ii) HRP detection system followed with DAB substrate or vector VIP substrate staining. Olympus Fluoview Viewer or Aperio Imagescope was used to image and quantify the fluorescence/chromogen signals in brain sections. As a control, the primary antibody was omitted from several test sections in each experiment. For Alexa-conjugated secondary antibodies, the sections were counterstained with Hoechst dye before mounting.

### Stereotaxic Injections of tetrapeptide inhibitor into mouse brains

Stereotaxic surgery was carried out as follows. Mice were anesthetized with 6X ketamine (0.1 mL/10 g body weight), depilated, given local anesthesia (lidocaine 0.3 mL), and placed securely in the ear bars. Intracerebroventricular (ICV) injections were given to mice (bregma coordinates: anterior/posterior +0.3, medial/lateral −1.0, dorsal/ventral −3.0) with 10.3 μl of 10 mM stock to inject 50 μg of tetrapeptide with 5 μg of PFF, at an infusion rate of 60 nl/ min. After the injection, the needle was left inserted for 5 min, then slowly withdrawn, and the scalp was securely stitched. Postoperative analgesia and antibiotic were administered intramuscularly once reflexes returned (septazol 0.05 mL, ketapro 0.05 mL) and mice were returned to their home cages. Mice were left to recover for 24 h. After 30 days, brains were removed from the skull, and processed for cryosectioning or western blot.

### Bimolecular Fluorescence Complementation (BiFC) Assay

*CRBN*^*+/+*^ and *CRBN*^*-/-*^ SH-SY5Y cells were seeded on coverslips in complete medium in 6-well plates (1 × 10^4^ cells/well). Cells were co-transfected with α-SYN-VN173 and α-SYN-VC155 constructs. After 16 h, cells were treated with MPTP for 48 h. The cellular intensities of α-SYN -BiFC fluorescence were analyzed using an FV1000 confocal laser scanning microscope (Olympus).

### Turbidity measurements

Turbidity instigated by α-SYN aggregation was examined with absorbance at 500 nm. The absorbance of each solution was monitored in a Beckman DU 7500 spectrophotometer.

### α-SYN pre-formed fibril (PFF) treatment

Recombinant α-SYN stocks (5 mg/ml) were used to generate PFF by putting them on an orbital shaker for seven days at 1000 rpm and 37 °C. PFF samples were stored at −80 °C until needed. Prior to treatment, PFF stocks were sonicated (30% Amplitude, 1 s alternating on/off pulse, 60 s total) after being diluted to 500 ug/mL in DPBS. Sonicated fibrils (10 µg/mL) were used to treat differentiated dopaminergic-like neurons.

### Protein expression and purification

Human Hsp70, Apg2, CRBN, DJ1, DJ2, DJ6, and α-SYN were expressed in E. coli BL21 (DE3) with an N-terminal His6-tag and purified by affinity chromatography on Ni-NTA affinity resin (Clontech). (IPTG 0.1 mM, Lysis buffer: 50 mM HEPES-KOH, 0.5 M NaCl. Wash buffer: 50 mM HEPES-KOH, 0.5 M NaCl, 50 mM imidazole. Elution buffer: 50 mM HEPES-KOH, 0.5 M NaCl, 0.5 M imidazole). Proteins were concentrated and frozen at −80 °C.

### α-SYN Fibril (PFF) formation

Purified α-SYN (200 µM) monomers were filtered through 0.2 µm filters and incubated in 1.5 ml-microfuge tubes at 37 °C, with orbital shaking (1000 rpm) at 37 °C for 1 week in buffer (50 mM sodium phosphate pH 7.3, 100 mM NaCl and 0.05% sodium azide).

### MTT assay

SH-SY5Y cells were plated at a density of 5 × 10^3^ cells/well in 96-well plates and transfected with different species of PFFs using Chariot^TM^ reagent and incubated for 48 h. MTT (3-(4,5Dimethylthiazol-2yl)-2,5-diphenyltetrazolium bromide) was added to the treated cells and incubated for 4 h. The quantity of formazan generated (indicative of cell viability) was measured by absorbance at 570 nm using a plate reading spectrophotometer.

### Tail Suspension test

The mice were suspended by the tail using a piece of adhesive tape. The tape was strong enough to prevent the mice from falling and did not damage the skin of the tails. The test persisted for 6 min. The total immobility time (time period during which the animal hangs passively and motionless) was measured for each animal, and reflected an index of “depression-like” behavior.

### Pole test

Mice were placed in the top of a 60 cm vertical pole having a diameter of 1 cm. The pole was mounted on a rectangular base stand kept in the home cage so that mice might choose to run down to the floor of cage. The time to turn completely downward and total time to descend to the floor were recorded.

### Synthesis of DJ1 tetra peptide

Rink amide MBHA resin (200 mg, 0.13 mmol, resin loading 0.65 mmol/g) was swelled in DMF (4 mL) at room temperature for 20 min. For Fmoc deprotection, the resin was treated with 2% (v/v) DBU in DMF (4 mL) at room temperature for 5 min. The resin was washed with CH_2_Cl_2_ (×2), DMF (×2), MeOH (×1), DMF (×2), and CH_2_Cl_2_ (×2), sequentially.

Next, peptide coupling was performed by the addition of the desired amino acid (0.39 mmol, 3.0 equiv.), HATU (148.3 mg, 0.39 mmol, 3.0 equiv.), and DIPEA (134.4 mg, 1.04 mmol, 8.0 equiv.) in DMF (4 mL). The mixture was stirred at room temperature for 2 h, and washed using the above sequence. Fmoc deprotection and peptide coupling were repeated until the desired sequence was obtained.

After the desired sequence was obtained, *N*-terminal acetylation was performed by the addition of acetic anhydride (398.2 mg, 3.9 mmol, 30.0 equiv.), and DIPEA (504.0 mg, 3.9 mmol, 30.0 equiv.) in DMF (4 mL). The mixture was stirred at room temperature for 2 h.

The peptide was cleaved with TFA/TIS/CH_2_Cl_2_ 95:2.5:2.5 (v/v/v) at room temperature for 2 h. The volatiles were evaporated using a stream of N_2_ gas, and the crude peptide was dissolved in a 1:1 (v/v) water/acetonitrile solution, filtered, lyophilized, and purified by Waters preparative high-performance liquid chromatography (HPLC) system (2489 UV/visible detector, 2545 Quaternary HPLC pump, Fraction collector III, Waters) with a normal phase amide column (XBridge BEH Amide OBD Prep Column, 19 × 150 mm, 5 μm, Waters) at room temperature. The flow rate was set to 7 mL/min. A binary mobile phase system (A: deionized water + 0.1% TFA, B: ACN + 0.1% TFA) was used under following conditions: 5 min 90% of B, followed by a linear gradient to 10% B during 10 min. LC-MS analysis was performed using an Agilent LC-MS system (1260 Infinity LC and 6120 Quadrupole MS with API electrospray ion source, Agilent, Santa Clara, CA, USA). Measurements were carried out in positive electrospray ionization mode with a scan range of 500–2000 m/z.

### Experimental design and statistical analysis

All in vivo experiments were performed on age-matched WT control, CRBN-KO and 5XFAD mice. All data were generated by at least five replicates from independently performed experiments, and the total number of mice or tissues used per group is given in the figure legends. Male mice were used and the sample size (n) was determined based on the literature. All data are presented as mean ± SEM of “n” independent experiments. In all figures, bars and lines show mean and error bars show SEM. Data were analyzed by student’s *t* test for the comparison of two groups, or one-way ANOVA for multiple groups. Excel was used for statistical analysis. *p* < 0.05 were considered statistically significant. The *p* values were presented as *p < 0.05, **p < 0.01, ***p < 0.001. ns, no significant difference.

### Materials for the synthesis of peptide inhibitors

Reagents and solvents were purchased from commercial vendors and used without further purification. Rink amide MBHA resin, Fmoc-Asp(OtBu)-OH, Fmoc-Gly-OH, Fmoc-Arg(Pbf)-OH, Fmoc-Thr(tBu)-OH were purchased from Novabiochem (Darmstadt, Germany). *O*-(7-Azabenzotriazol-1-yl)-*N*,*N*,*N*’,*N*’-tetramethyluronium hexafluorophosphate (HATU) was purchased from Chem-Impex (Wood Dale, IL, USA). *N*,*N*-Diisopropylethylenediamine (DIPEA) and acetic anhydride were purchased from Tokyo Chemical Industry (Tokyo, Japan). 1,8-Diazabicyclo[5.4.0]undec-7-ene (DBU) and triisopropylsilane (TIS) were purchased from Sigma-Aldrich (St. Louis, MO, USA). Trifluoroacetic acid (TFA) and *N*,*N*-dimethylformamide (DMF) (≥99.9%, for peptide synthesis grade) were purchased from Acros Organics (Fair Lawn, NJ, USA). Acetonitrile (HPLC grade) and dichloromethane (HPLC grade) were purchased from Thermo Fisher Scientific (Waltham, MA, USA). Containers for peptide synthesis (empty cartridge, frit, and cap plugs) were purchased from Applied Separations (Allentown, PA, USA).

## Supplementary information

Supplementary material

## Data Availability

The authors declare that all data supporting the findings of this study are available within this article and its supplementary information file.
