# Supplementary material for: CRBN modulates synuclein fibrillation via degradation of DNAJB1 in mouse model of Parkinson disease
Source: NPJ Parkinsons Dis. 2024 Oct 23;10:194. doi: 10.1038/s41531-024-00801-3 (PMC11500381; doi:10.1038/s41531-024-00801-3)
Supplement: Supplementary file 1 — Supplementary material [file 41531_2024_801_MOESM1_ESM.pdf]

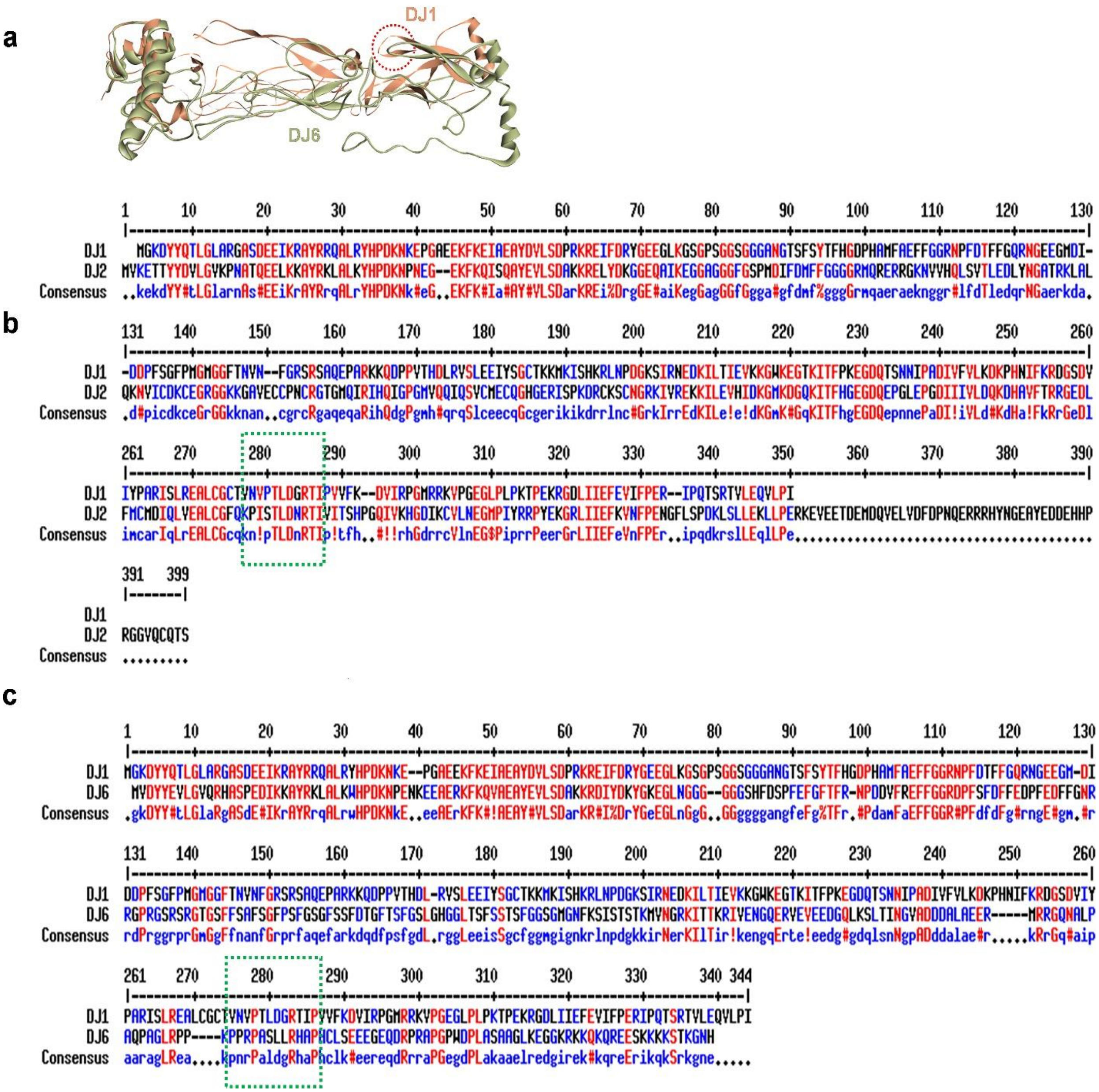

Supplementary Fig. 1: DJ1 and DJ6 exhibit structural homology with DJ2.

(a) DJ1 has structural similarity with DJ6. 3D models were generated with I-TASSER server. **(b and c)** J-domain of DJ1 has sequence similarity with DJ2 **(b)** and DJ6 **(c)**. Binding loop of DJ2 is well conserved in DJ1 but not in D6 as shown in the highlighted boxes.

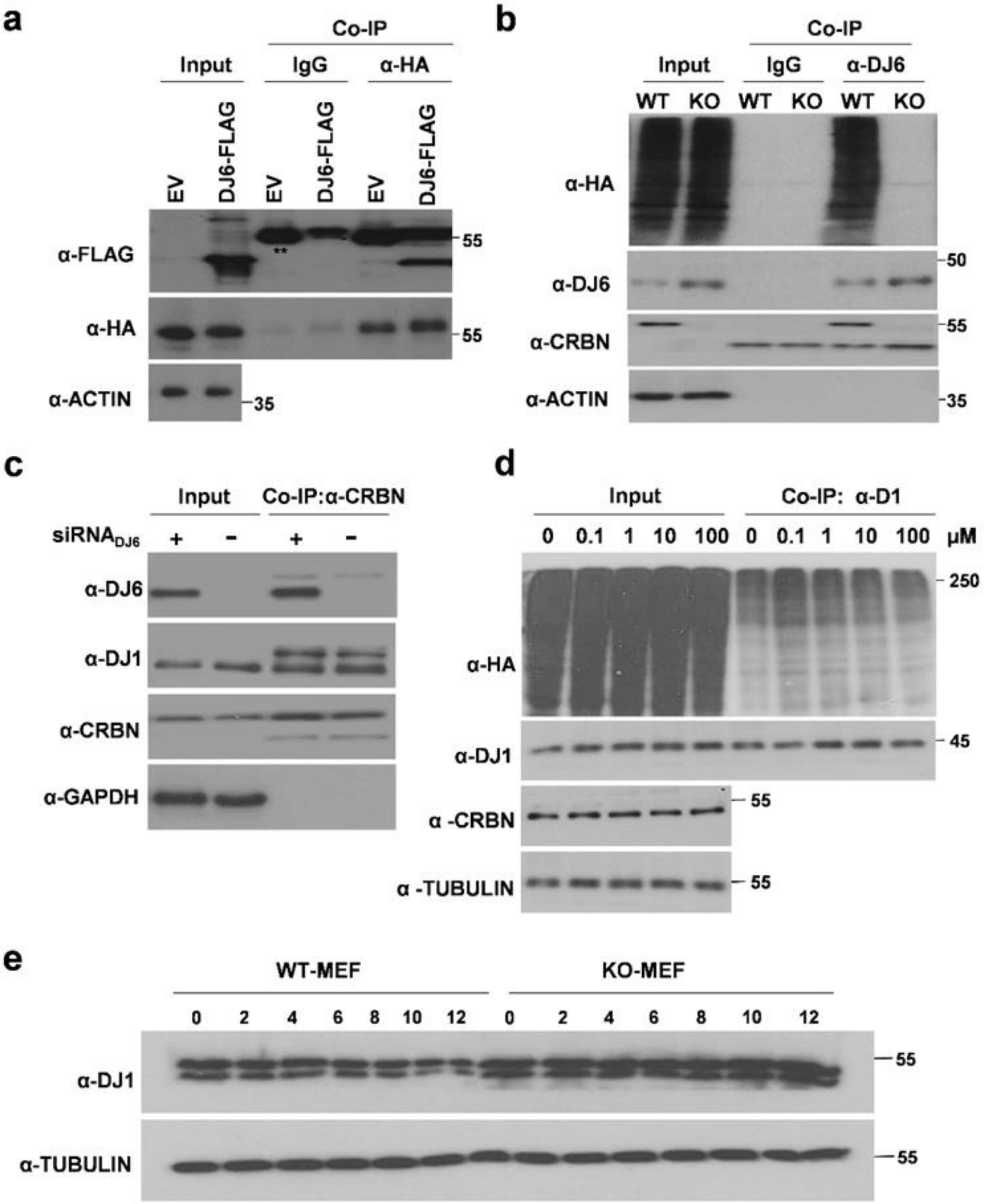

Supplementary Fig. 2: Ubiquitination of DJ1 is not affected by thalidomide.

(**a**) SH-SY5Y cells were transiently transfected with FLAG-D6 and HA-CRBN. Co-IP was performed with α-HA antibody and blotted with the indicated antibodies. **(b)** SH-SY5Y cells were transiently transfected with HA-Ub and lysed in Ubn buffer, IP was performed with α-DJ6 and immunoblotting was performed with the indicated antibodies. **(c)** SH-SY5Y cells were

transiently transfected with siRNA targeting DJ6 and Co-IP was performed with α-CRBN antibody and blotted with the antibodies mentioned. **(d)** SH-SY5Y cells were transiently transfected with HA-Ub and lysed in Ubn buffer, IP was performed with α-DJ1 after treating the cells with thalidomide. **(e)** WT (*Crbn*$^{+/+}$) and KO (*Crbn*$^{-/-}$) MEF cells were treated with 2.5 µg/ml CHX or 0.5 µg/ml MG132 respectively and harvested at the indicated time points.

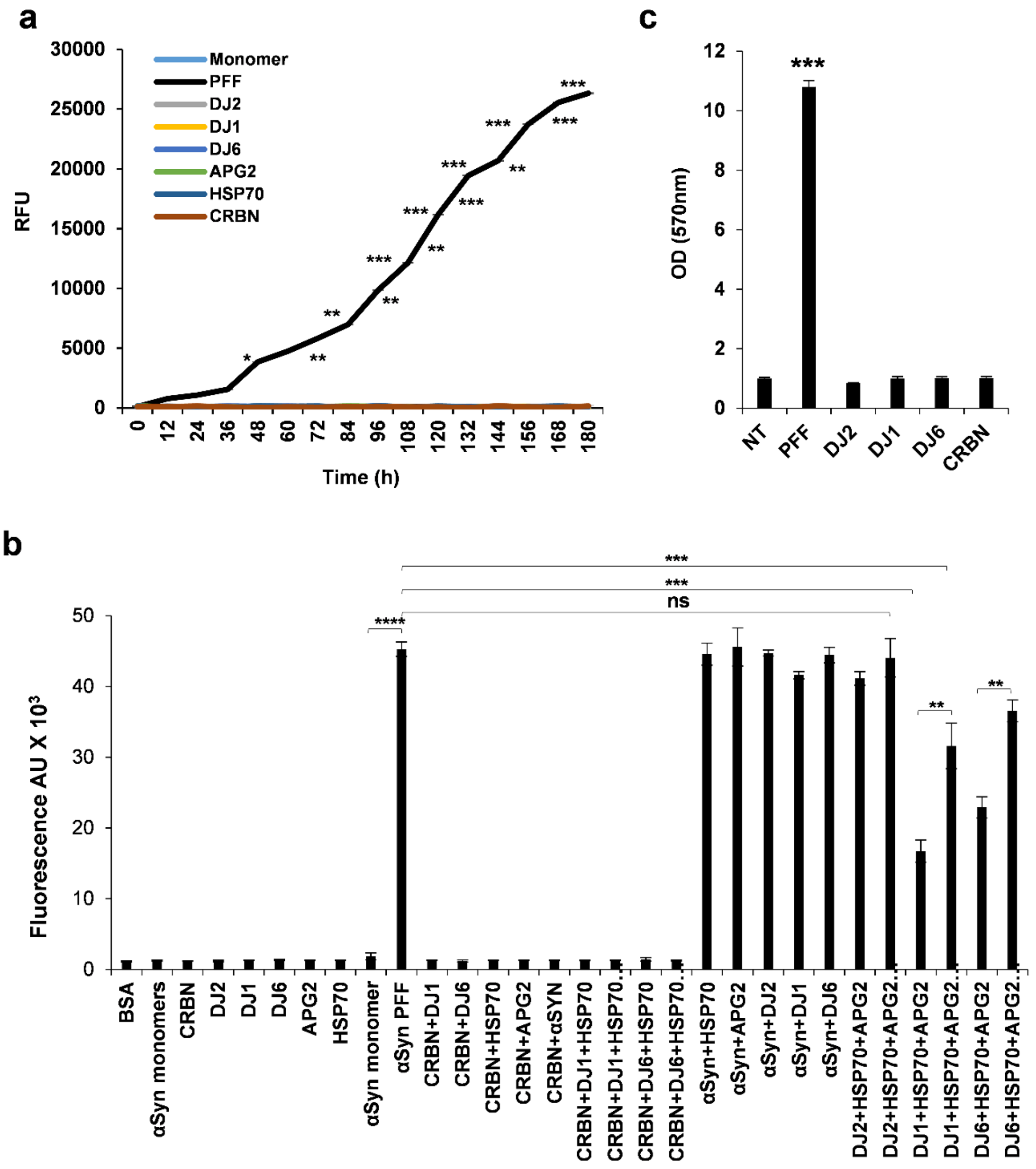

Supplementary Fig. 3: Control experiments for the aggregation of α-SYN.

(a, b) To monitor the effects of recombinant proteins on the fluorescence, full-length α-SYN and the indicated proteins were incubated at 37 ºC for 180 hr **(a)** or with the different combinations indicated **(b)**. Samples were collected at the time points mentioned and kept at -80 ºC until the collection of all samples. 5 μM ThT was added after to observe the degree of aggregation and fluorescence emission was measured at 480 nm, with excitation at 440 nm. **(c)** Differentiated SH-SY5Y cells were treated with monomeric (named as non-treated NT), PFFs, DJ2, DJ1, DJ6 and CRBN as shown in the figure. MTT assay was performed after 48 h to assess cell viability.

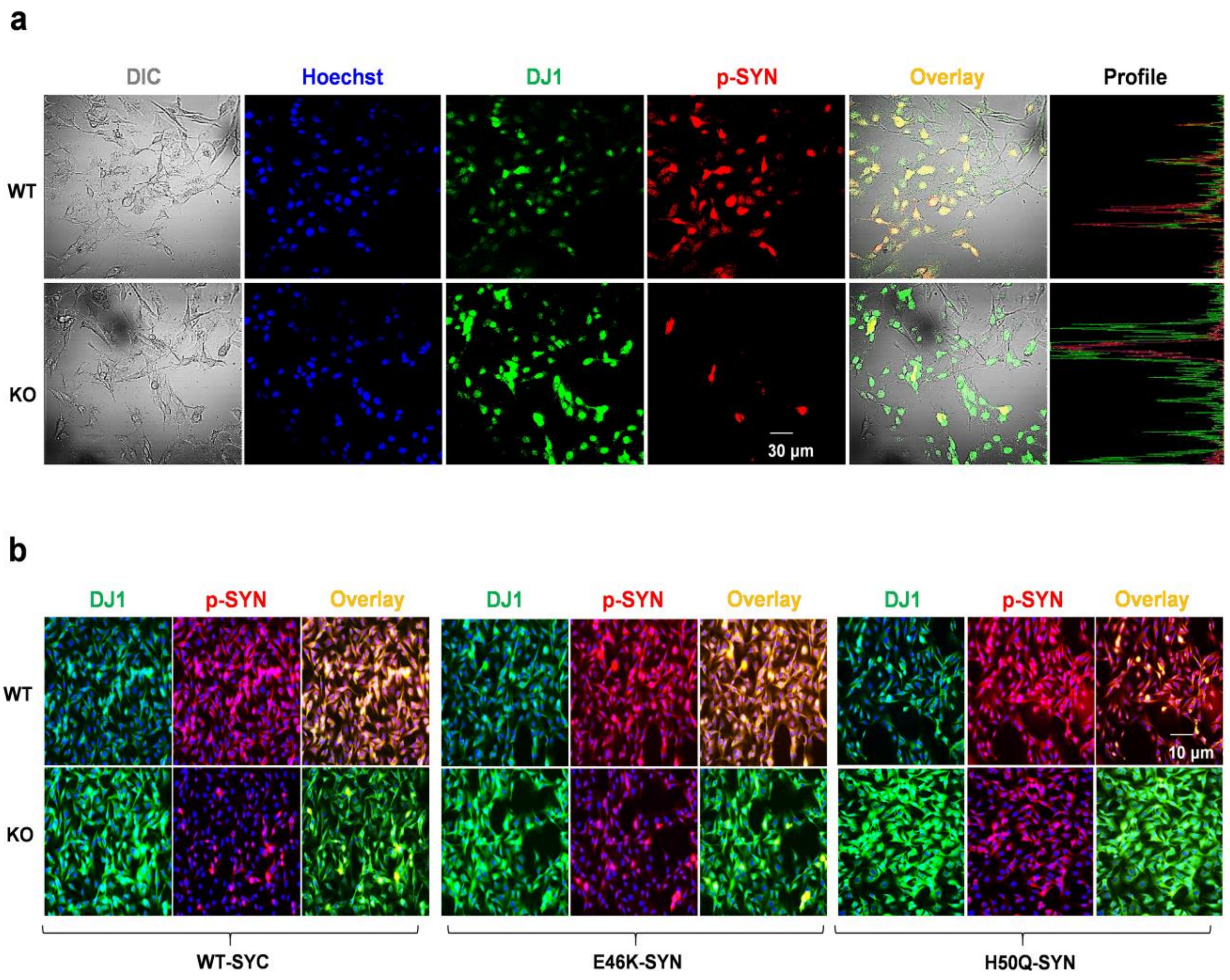

Supplementary Fig. 4: ***CRBN*** $^{-/-}$ **(KO) cells exhibit resistance toward the toxicity of α-SYN mutants.**

(a) Differentiated *CRBN* $^{+/+}$ (WT) and *CRBN* $^{-/-}$ (KO) SH-SY5Y were analyzed by immunocytochemistry to measure the expression of DJ1 and phosphorylated α-SYN (p-SYN).

(b) WT and KO SHSY5Y cells were transfected with WT or mutant forms of α-SYN as mentioned. DJ1 and p-Synuclein levels were measured by immunocytochemistry after 48h. The cells were imaged with a fluorescence microscope. n = 5.

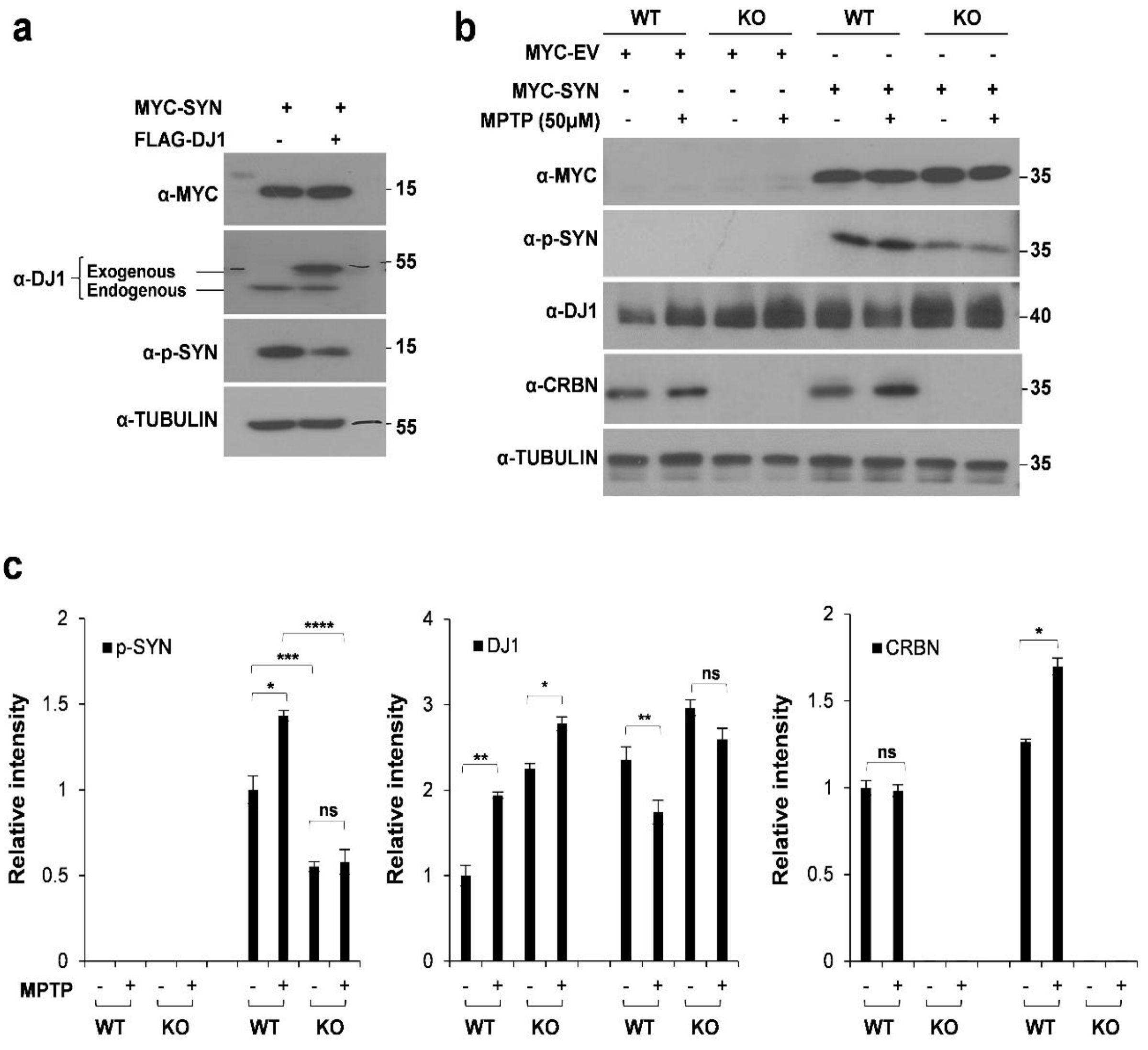

Supplementary Fig. 5. CRBN tunes the chaperone activity of DJ1 to prevent synuclein phosphorylation.

(a) SH-SY5Y cells were transiently co-transfected Myc-SYN and FLAG-DJ1 and phosphorylated α-SYN was measured. **(b)** SH-SY5Y cells were subjected to CRISPR/Cas9-mediated knock out (KO) of *CRBN* or non-targeting gRNA sequences (NC or negative control vector). Myc-SYN was transiently transfected to both cell lines and western blot was performed with the indicated antibodies. **(c)** Statistical analysis of panel **(b)**.

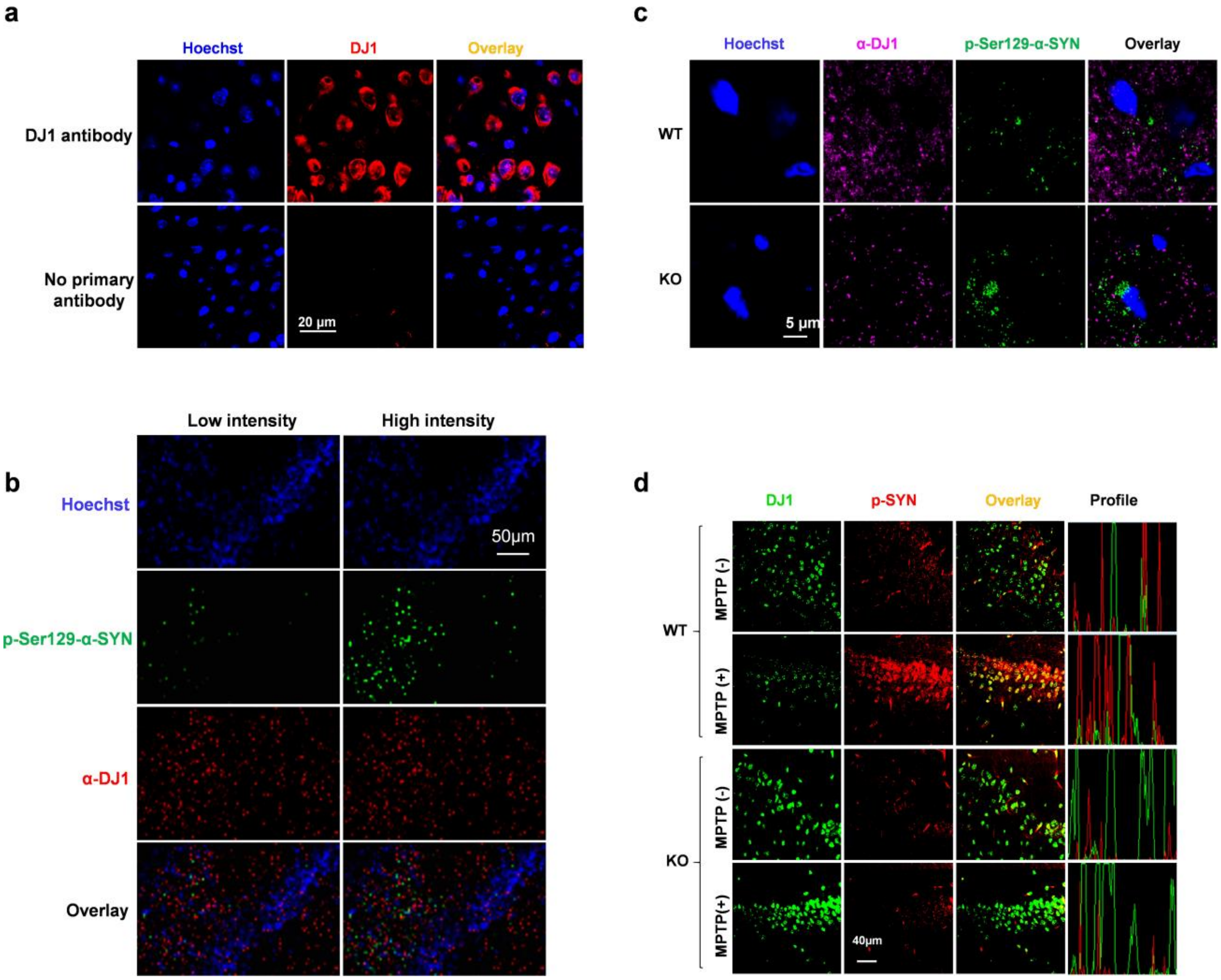

Supplementary Fig. 6. Immunohistochemistry analysis of brains from WT and KO mice. **(a)** The control samples of immunohistochemistry without primary antibodies in the granular region of the hippocampus. **(b)** IHC imaging of hippocampus with low and slightly increased intensity of laser for p-Ser129-α-SYN signals. **(c)** The magnified images were taken for IHC staining at 100X to make sure that the immunostaining is consistent for WT and KO samples rather than being parenchymal or intracellular for different samples**. (d)** Levels of p-Ser129-α-SYN and DJ1 after intraperitoneal injection of MPTP to WT and KO mice.

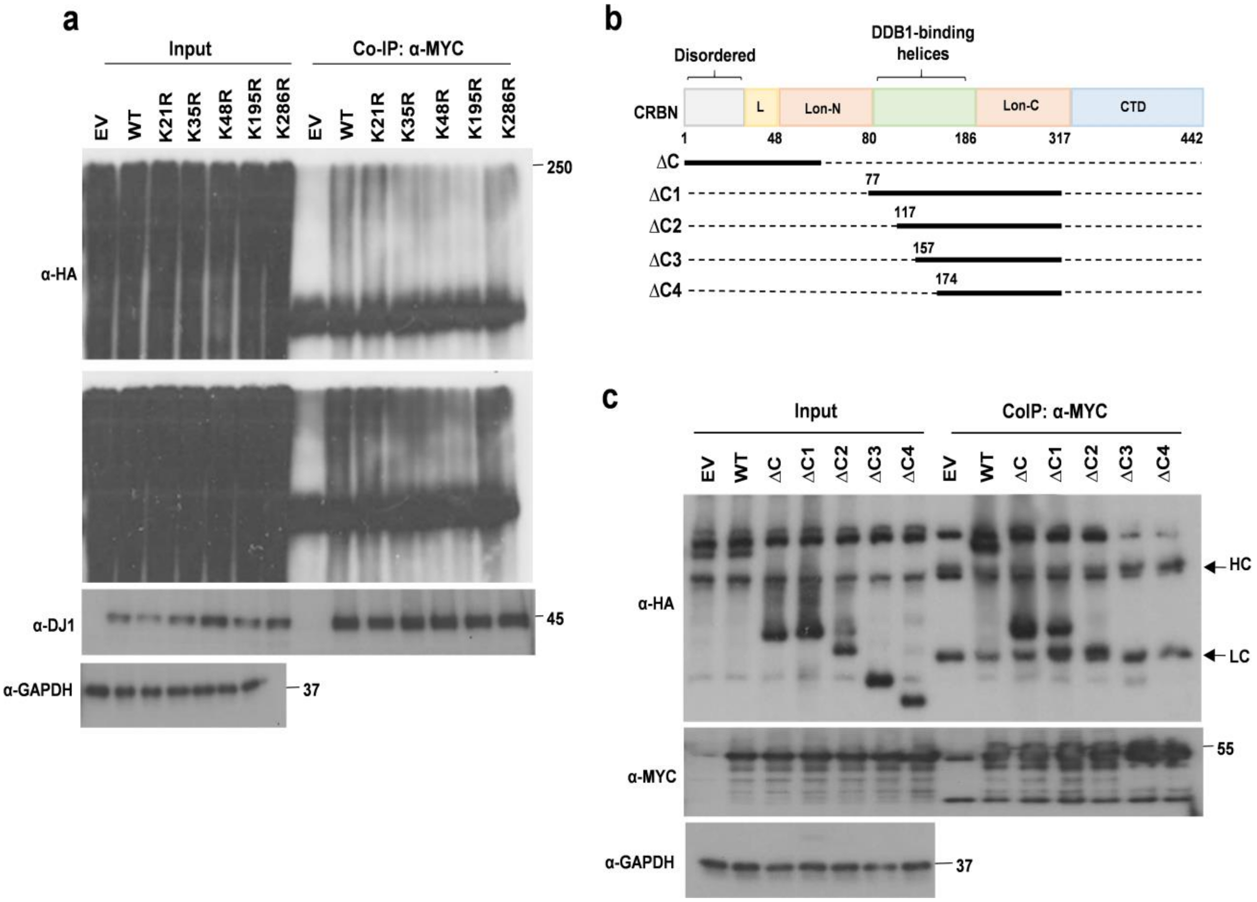

Supplementary Fig. 7. Localization of specific lysine residues responsible for the ubiquitination of DJ1.

(a) The PhosphositePlus database was used to identify the experimentally confirmed lysines ubiquitinated in DJ1. SH-SY5Ycells were transfected with empty vector (EV), wild-type (WT) DJ1 or various lysine mutants and HA-Ub was co-transfected. Ubiquitination assay was performed as described in materials and methods. **(b)** Schematic diagram of the rCRBN constructs with HA-tag. L (linker), CTD (C-terminal domain), Lon-N (N-terminal Lon domain), Lon-C (C-terminal Lon domain). **(c)** SH-SY5Y cells were transiently co-transfected with the genetic constructs indicated in panel **(b)**. Cellular extracts were immunoprecipitated with the indicated antibodies. A band at ~55 kDa represents IgG heavy chains (HC) and at ~25 kDa represents IgG light chains (LC).

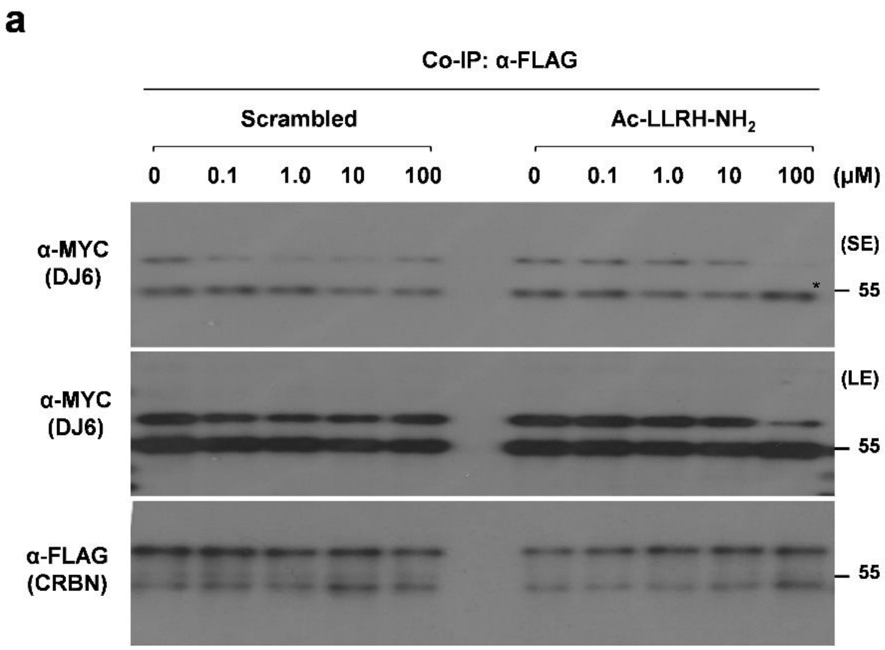

Supplementary Fig. 8: Effect of tetrapeptide inhibitor to inhibit the selective activity of CRBN for DJ6.

(a) SH-SY5Ycells were transfected with Myc-DJ6 and FLAG-CRBN. Cells were lysed and incubated with scramble (RHLL) or DJ6-based tetrapeptide inhibitor LLRH with both ends blocked (Ac-LLRH-NH_2). CoIP was performed with α-FLAG antibody.

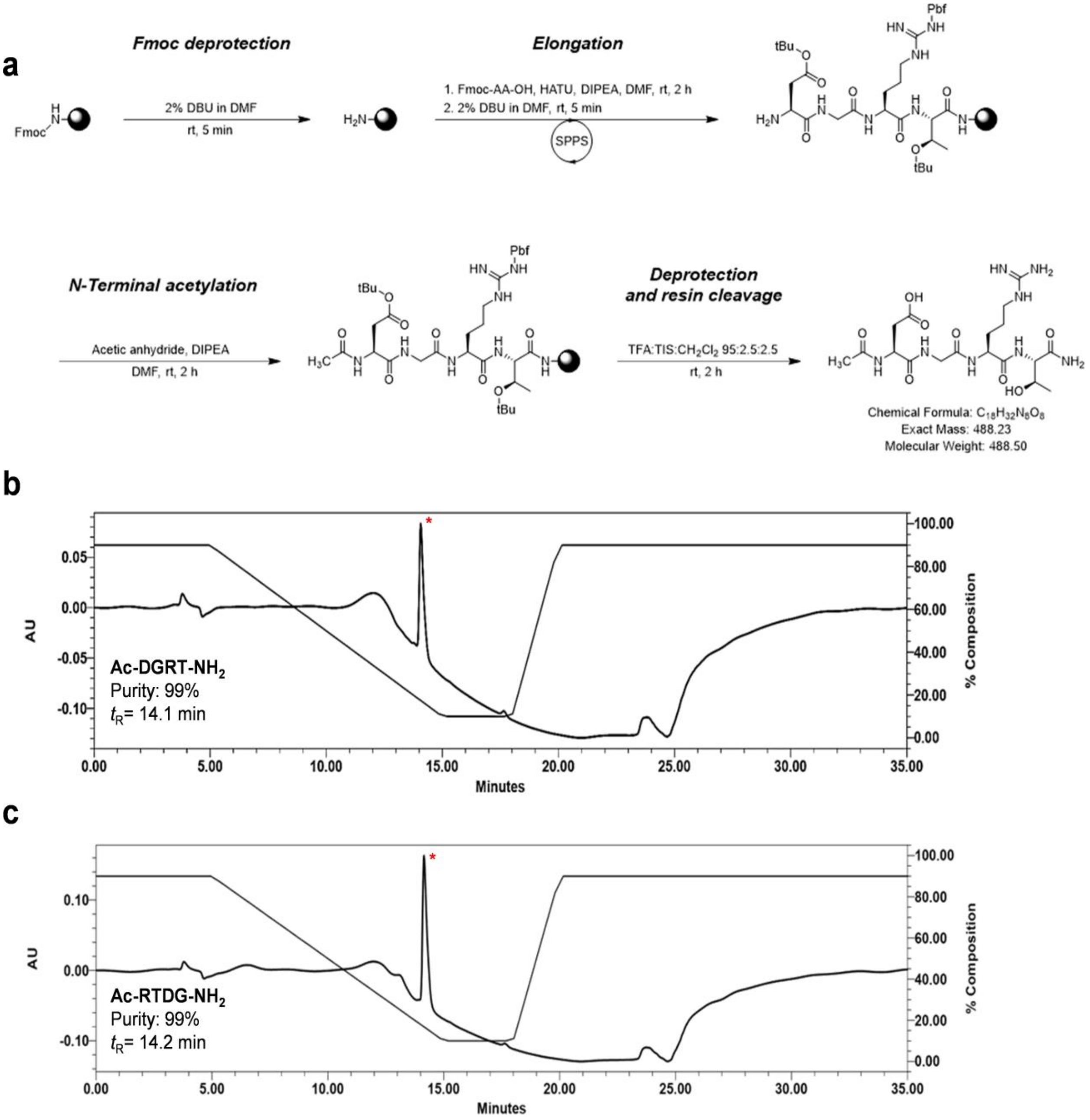

Supplementary Fig. 9: Synthesis of tetrapeptide inhibitor to ablate the selective activity of CRBN for DJ1.

(A) Schematic representation of synthesis of tetra peptide inhibitors. **(b, c)** HPLC chromatogram of the DJ1 tetra peptide Ac-DGRT-NH_2 **(b)** and scrambled peptide **(c)** monitored at 220 nm.

a

b

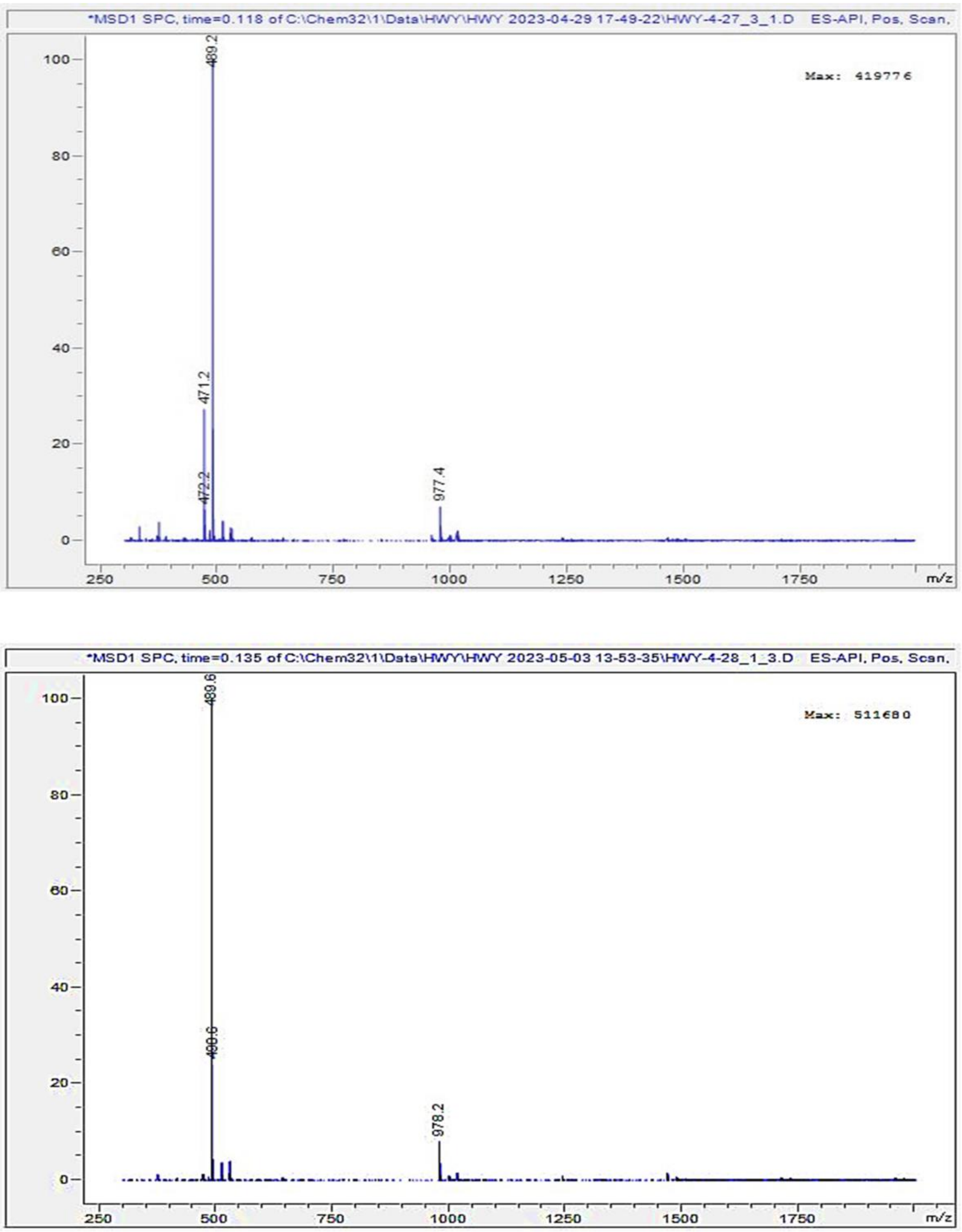

Supplementary Fig. 10: ESI-MS spectrum of DJ1 tetra peptide.

ESI-MS spectrum of the DJ1 tetra peptide Ac-DGRT-NH_2 **(a)** and scrambled peptide Ac-RTDG-NH_2 **(b)**.

a

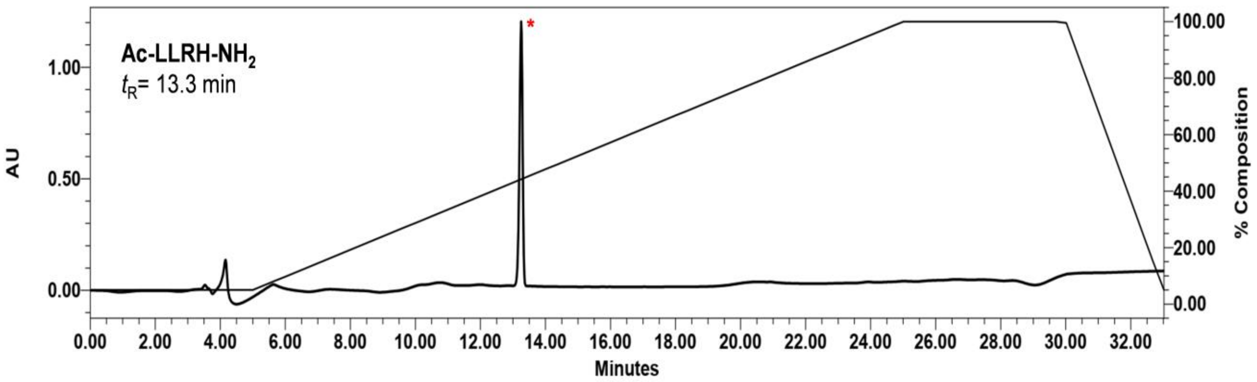

HPLC chromatogram of DJ6 tetra peptide (Ac-LLRH-NH_2) monitored at 220 nm.

b

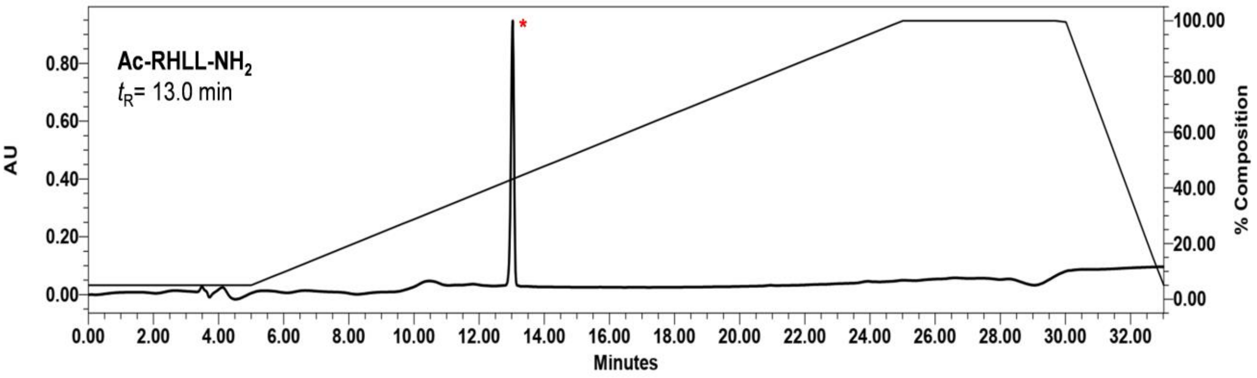

HPLC chromatogram of scrambled peptide (Ac-RHLL-NH_2) monitored at 220 nm.

Supplementary Fig. 11: HPLC chromatograms of DJ6 tetra peptide.

HPLC chromatograms **(a and b)** of the DJ6 tetra peptide Ac-LLRH-NH_2 and scrambled peptide Ac-RHLL-NH_2.

SUPPLEMENTARY TABLES:

Supplementary Table 1:

Significance levels (*p*-values) are shown for **d, e** and **f** panels of Figure 2 of manuscript.

TIME	Figure 2d				Figure 2e			Figure 2f		
	Compared to monomers	Compared to PFF		Compared to PFF+DJ2	Compared to PFF	Compared to PFF+DJ1		Compared to PFF		Compared to PFF+DJ6
	**PFF**		**PFF+DJ2**	**PFF+ DJ2+CRBN**		**PFF+DJ1**	**PFF+ DJ1+CRBN**		**PFF+DJ6**	**PFF+ DJ6+CRBN**
**0h**	0.55432201		0.74854089	0.5312199		0.1882801	0.45025878		0.28861964	0.07236285
**12h**	0.00212003		0.0837827	0.29243362		0.00239801	0.00388374		0.00555003	0.00050163
**24h**	0.00022977		0.00490401	0.1087204		0.00014427	3.589E-06		0.00036584	3.0485E-06
**36h**	0.00058232		0.0088946	0.00016821		0.00134769	1.6133E-05		0.00195141	3.9282E-08
**48h**	1.5237E-05		0.00077933	0.18723575		3.6518E-05	9.6107E-05		0.00032328	0.00034755
**60h**	8.3008E-05		0.04784511	0.00838022		0.00018631	1.4223E-05		0.00024259	1.9934E-06
**72h**	4.5978E-06		0.00804363	0.0275653		1.1981E-05	8.015E-05		2.0108E-05	0.00080026
**84h**	8.2535E-06		0.01307654	0.0195937		2.1267E-05	7.2975E-08		3.5956E-05	6.6421E-05
**96h**	3.052E-06		0.00028643	0.00499617		5.9428E-06	0.00020416		7.1887E-06	6.7463E-05
**108h**	3.4161E-05		0.00088322	0.00722366		7.3316E-05	2.5876E-05		7.9346E-05	0.00061628
**120h**	6.8485E-06		0.00045729	0.04113151		1.4554E-05	3.0134E-05		1.4948E-05	2.3468E-05
**132h**	1.0178E-06		0.00126006	0.34970222		3.1642E-06	0.00023758		2.5747E-06	7.9124E-05
**144h**	8.3899E-07		0.00285989	0.05351642		4.4124E-06	2.9552E-05		6.3328E-06	2.8943E-05
**156h**	1.9918E-07		0.00011847	0.02000071		2.8744E-06	1.2829E-05		1.3545E-06	3.473E-05
**168h**	1.9804E-07		0.00010019	0.00034607		8.7347E-07	2.3457E-06		1.4745E-06	2.3419E-07
**180h**	9.9407E-08		0.00038721	0.00220797		7.414E-06	1.23E-05		6.0964E-07	1.1148E-06

Supplementary Table 2.

ESI-MS m/z values of DJ1 and DJ6 tetra peptides and scrambled peptides (ESI-MS: Electrospray Ionisation Mass Spectrometry, m/z: Molecular weight / Charge)

**Compound**	**Molecular formula**	**Calc. *m/z*, $[M+H]^+$**	**Obs. *m/z*, $[M+H]^+$**
Ac-DGRT-NH_2	$C_{18}H_{32}N_8O_8$	488.23	489.2
Ac-RTDG-NH_2	$C_{18}H_{32}N_8O_8$	488.23	489.6
Ac-LLRH-NH_2	$C_{26}H_{46}N_{10}O_5$	579.37	579.3
Ac-RHLL-NH_2	$C_{26}H_{46}N_{10}O_5$	579.37	579.3

MATERIAL FOR TETRAPEPTIDE SYNTHESIS:

Reagents and solvents were purchased from commercial vendors and used without further purification. Rink amide MBHA resin, Fmoc-Asp(OtBu)-OH, Fmoc-Gly-OH, Fmoc-Arg(Pbf)-OH, Fmoc-Thr(tBu)-OH were purchased from Novabiochem (Darmstadt, Germany). *O*-(7-Azabenzotriazol-1-yl)-*N*,*N*,*N*',*N*'-tetramethyluronium hexafluorophosphate (HATU) was purchased from Chem-Impex (Wood Dale, IL, USA). *N*,*N*-Diisopropylethylenediamine (DIPEA) and acetic anhydride were purchased from Tokyo Chemical Industry (Tokyo, Japan). 1,8-Diazabicyclo[5.4.0]undec-7-ene (DBU) and triisopropylsilane (TIS) were purchased from Sigma-Aldrich (St. Louis, MO, USA). Trifluoroacetic acid (TFA) and *N*,*N*-dimethylformamide (DMF) (≥99.9%, for peptide synthesis grade) were purchased from Acros Organics (Fair Lawn, NJ, USA). Acetonitrile (HPLC grade) and dichloromethane (HPLC grade) were purchased from Thermo Fisher Scientific (Waltham, MA, USA). Containers for peptide synthesis (empty cartridge, frit, and cap plugs) were purchased from Applied Separations (Allentown, PA, USA).
